# Inferring Infection Patterns Based on a Connectivity Map of Host Transcriptional Responses

**DOI:** 10.1038/srep15820

**Published:** 2015-10-28

**Authors:** Lu Han, Haochen He, Fei Li, Xiuliang Cui, Dafei Xie, Yang Liu, Xiaofei Zheng, Hui Bai, Shengqi Wang, Xiaochen Bo

**Affiliations:** 1 Department of Biotechnology, Beijing Institute of Radiation Medicine, Beijing, 100850, China; 2 Department of Traditional Chinese Medicine and Neuroimmunopharmacology, Beijing Institute of Pharmacology and Toxicology, Beijing, 100850, China; 3 International Cooperation Laboratory on Signal Transduction, Eastern Hepatobiliary Surgery Institute, Second Military Medical University, Shanghai, 200433, China; 4 Department of Biochemistry and Molecular Biology, Beijing Institute of Radiation Medicine, Beijing, 100850, China; 5 Department of Pharmacy, No.451 hospital of People’s Liberation Army, Xi’an, 710065, China

## Abstract

Host responses to infections represent an important pathogenicity determiner, and delineation of host responses can elucidate pathogenesis processes and inform the development of anti-infection therapies. Low cost, high throughput, easy quantitation, and rich descriptions have made gene expression profiling generated by DNA microarrays an optimal approach for describing host transcriptional responses (HTRs). However, efforts to characterize the landscape of HTRs to diverse pathogens are far from offering a comprehensive view. Here, we developed an HTR Connectivity Map based on systematic assessment of pairwise similarities of HTRs to 50 clinically important human pathogens using 1353 gene-expression profiles generated from >60 human cells/tissues. These 50 pathogens were further partitioned into eight robust “HTR communities” (i.e., groups with more consensus internal HTR similarities). These communities showed enrichment in specific infection attributes and differential gene expression patterns. Using query signatures of HTRs to external pathogens, we demonstrated four distinct modes of HTR associations among different pathogens types/class, and validated the reliability of the HTR community divisions for differentiating and categorizing pathogens from a host-oriented perspective. These findings provide a first-generation HTR Connectivity Map of 50 diverse pathogens, and demonstrate the potential for using annotated HTR community to detect functional associations among infectious pathogens.

In recent years, there has been a growing recognition of the importance of host responses to pathogenic infection in characterizing microbial pathogenesis, disease diagnosis, and prognosis, as well as for novel therapy development^1^. Host transcriptional responses (HTRs) depicted by gene expression profiles are practical technically and can provide a refined description of the complexity of pathogenic infection and disease states with wide coverage and excellent discrimination^2^.

In principle, systematic comparative analyses of host cell responses to a variety of pathogens have the potential to be a fruitful means of disentangling host-pathogen interactions^3^. Hierarchical clustering has been used extensively to integrate and analyze profile data with the aim of identifying novel genetic factors and complex host cellular defense mechanisms involved in particular types of infection^4^,^5^,^6^,^7^,^8^. Indeed, with this clustering method, common HTRs to microbial infections have been identified and the resultant data have been used to identify host-oriented broad-spectrum drug targets^9^. However, the approach yields a fairly limited and narrow slice of information. In most cases, only the most differentially dysregulated genes (i.e., signature genes) are subjected to systematic annotation in a focal analysis. The overlaps between HTR signature genes identified in studies thus have been far too small to allow broad scale examination of HTRs across pathogens.

Inspired by the Connectivity Map^10^ developed to summarize functional connections among a variety of small-molecule drugs, we developed an HTR Connectivity Map (Fig. 1). Our aim was to establish landscape associations across various pathogens based on an objective assessment of HTR similarities using a combination of transcriptional bioinformatics methods. Briefly, we gathered 1,353 reference gene expression profiles from more than 60 human cells/tissues infected with 50 clinically important pathogen types and then implemented an unbiased HTR characterization strategy and rank-based expression profile comparisons^11^,^12^ to evaluate 1,225 pairwise pathogen-pathogen HTR similarities (Fig. 1). We further divided these first 50 pathogens into groups with significant internal HTR similarity and characteristic modes of host gene expression patterns tagged with specific infection attributes, i.e., a reference resource known as HTR community. The annotations for community pathogens allowed us to propose, with an unprecedented host-oriented perspective, new associations for well-known pathogen taxonomy classes and novel associations for microenvironment-related and clinically relevant pathogens among these 50 infectious pathogens (Fig. 1).

Using HTR signatures from external pathogens, we provided in the present study proof-of-concept evidence that HTR community scheme can be used to (i) recognize pathogen class related to common featured HTRs (e.g., proteobacteria), (ii) discern the pathogenicity of pathogens with close phylogenetic relations (e.g., *Streptococcus* species), (iii) identify HTRs that are representative of particular microbiota and reflect a degree of host adaptation (e.g., oral commensal *vs*. pathogenic bacteria), and (iv) discover unknown common and unique HTRs to pathogens whose infections produce similar clinical presentations (e.g., respiratory viruses).

## Results

### First-generation HTR Connectivity Map

#### Reference profiles

After preliminary screening (see Methods), 82 gene expression datasets (including 1,353 gene expression profiles representing 893 infection-control pairs) encompassing HTRs to 50 pathogens (21 bacterial, 23 viral, 5 protozoan, and 1 fungal) were collected cumulatively (Supplementary Table S1). These 50 pathogens represent a broad range of clinically important pathogenic (sub)types and strains, and each was tested with its primary target tissue/cell tropism.

The HTRs of specific cell types to infection with particular pathogens were collated in a phenotype rank list (PRL) (infection-control matching strategy detailed in the Methods). And the Spearman correlations between each PRLs were presented as a heat map (Supplementary Figure S1). The Spearman correlations (mean value = 0.1164) between PRLs of a specific pathogen type were significantly higher than those (mean value = 0.0108) across different pathogen types (*P* < 10^−100^, two-sample *t*-test), and the corresponding area under curve (AUC) in the receiver operating characteristic (ROC) curve was 0.6625 (Supplementary Figures S2 and S3). The mean Spearman correlation coefficient for comparisons between PRLs of the same cell types across infections by different pathogens was 0.0349, with an AUC of 0.5614 (Supplementary Figures S2 and S4). Therefore, HTRs of a specific cell type had relatively weak correlations across different pathogens compared to HTRs of different cell types infected with the same pathogen (Supplementary Figures S1–S4 and Supplementary Data S1).

Employing a hierarchical majority-voting scheme^12^,^13^, we developed merged PRLs (mPRLs) (Supplementary Data S2) for each pathogen’s HTRs. We further proved that the mPRLs captured the consensus and common transcriptional responses to pathogens across settings (i.e., pathogen strain/subtype, infected cell line and laboratory) (Supplementary Figure S5 and Supplementary Data S3).

#### Fifty-pathogen HTR Connectivity Map

The 250 top- and bottom-ranked genes of each PRL for the signature HTRs for each pathogenic infection were selected (Supplementary Figure S6 and Supplementary Data S4; size of 250 based on estimated influence of signature size as detailed in the Methods). The pathogen-to-pathogen HTR connections were represented as an “association score” and computed with a PRL comparing method based on gene set enrichment analysis (GSEA)^10^,^11^,^14^.

**Figure 2:**
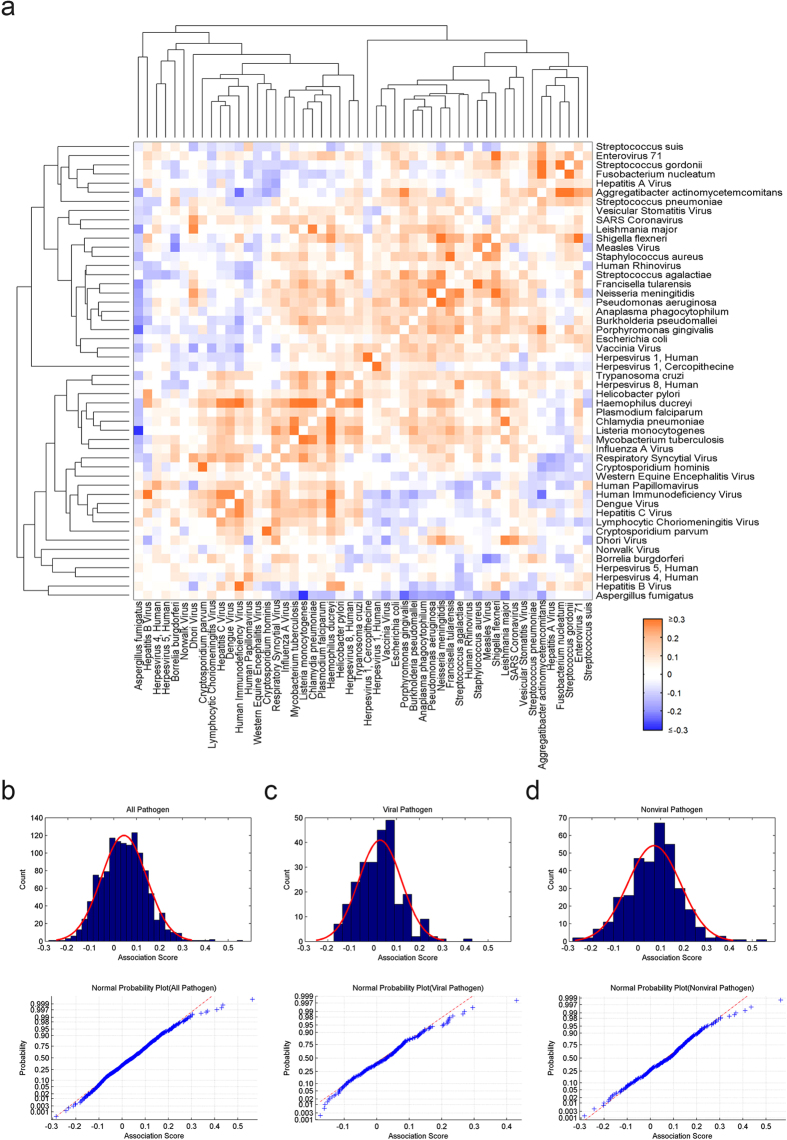
HTR Connectivity Map of 50 pathogens. (**a**) Heat map representation of association scores for HTR connectivities among 50 pathogens (a heat map of association scores calculated based on individual pathogen strain is in Supplementary Figure S1). The association scores among these 50 mPRLs are reported with corresponding *P* values and FDR values in Supplementary Data S5. The color of each cell represents the HTR connectivity association score computed for the mPRLs of each pathogen pair, with red representing positive connectivity and blue representing negative connectivity. Distributions of association scores for 1,225 pairs of HTRs among (**b**) all 50 mapped pathogens, (**c**) the 27 mapped non-viral pathogens (21 bacterial species, 5 protozoans, and 1 fungus), and (**d**) the 23 mapped viral pathogens, as approximated to normal distributions. All distribution patterns were in accordance with Gaussian distribution, and presented as means with standard deviations, i.e., 0.0447 ± 0.0987 (50 pathogens), 0.0711 ± 0.1153 (non-viral pathogens), and 0.0278 ± 0.0931 (viral pathogens). All distributions deviated from their expected zero centers (*P* = 7.67 × 10^−52^, 1.07 × 10^−26^, and 1.70 × 10^−6^ for all 50, non-viral subset, and viral subset, respectively, two-sample *t*-tests).

A heat map (Fig. 2a) was produced from 1,225 pairs of HTR connections among these 50 pathogens (Supplementary Data S5). The association scores had a Gaussian distribution with a mean value (0.0447) that differed significantly from zero (Fig. 2b; *t*-test *P* = 7.67 × 10^−52^), indicating a tendency for similar HTRs across infections. Meanwhile, HTR similarity for non-viral pathogens (mean = 0.0711) was greater than that for viral pathogens (mean = 0.0278) (Fig. 2c,d; two-sample *t*-test *P* = 2.25 × 10^−7^).

To identify infection attributes underlying HTR similarities across pathogen pairs, we collected the following four categories of information for each pathogen: (i) Medical Subject Headings (MeSH)^15^ biological classification code; (ii) infection-affected organ/tissues/cell(s); (iii) manifestation of infectious disease; and (iv) other literature-based laboratory and clinical characteristics (Supplementary Tables S1 and S2). Infection attribute labeling uncovers crucial factors underlying HTR similarity, while validating its reliability. We found that HTR similarity was not simply closely related to the benchmarks for each of the above attribute categories (Supplementary Figure S7a).

To identify individual infection attributes that associate strongly with particular significant HTR similarities between pathogen pairs, we calculated HTR-attribute association scores (range, −1 to +1), and obtained permutation *P* values through comparison with those in random trials using a Kolmogorov-Smirnov statistic-based approach. The association scores for the similarity features of taxonomy, cell tropism, infectious disease, and laboratory/clinical characteristics were 0.181, 0.167, 0.213 and 0.240, respectively (permutation *P* = 0.06, 0.16, 1 × 10^−5^, and 7 × 10^−5^, respectively). Thus, disease manifestation and laboratory/clinical characteristic similarities associated significantly (*P* < 0.01) with HTR similarity. Calculation of each infection attribute’s HTR-association score and corresponding permutation *P* value (minimum, five-pathogen commonality to assure the reliability; threshold false discovery rate (*FDR*) < 0.01), revealed 23 highly HTR-associated infection attributes (Supplementary Table S3), including intracellular infection, commensalism, Gram-negative bacteria, droplet contact transmission, non-motile bacteria, and manifestation of lung diseases (Supplementary Figure S7b).

#### HTR communities

**Figure 3:**
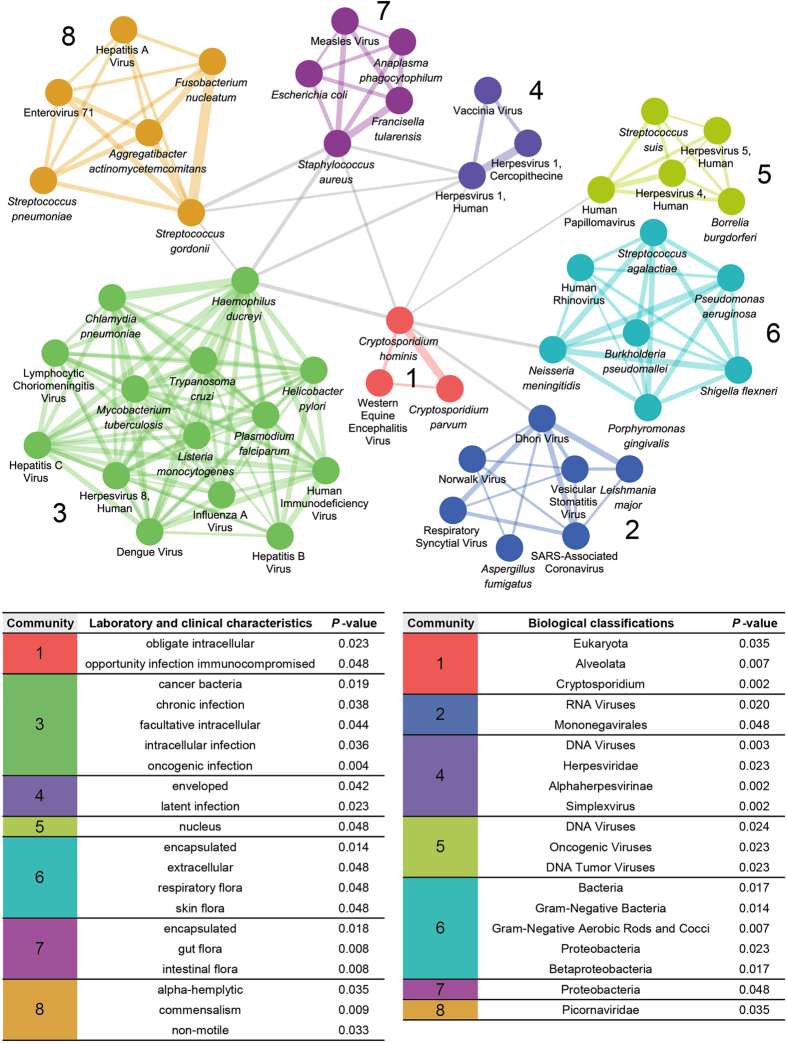
HTR communities. Each node represents a pathogen type. Pathogen pairs whose HTRs were significantly similar are connected with an edge, the thickness of which is proportional to the pair’s association score. A community is defined as a group of nodes that are closely interconnected with each other, with fewer connections to nodes outside the group. HTR communities were identified based on association scores and labeled numerically according to the alphabetical precedence of the exemplar pathogen. Enriched HTR-related biological classifications, along with laboratory and clinical characteristics, are summarized (detailed information in Supplementary Table S4).

Application of an automated, parameter-free clustering algorithm^16^ yielded eight pathogen groups with prominent consensus internal HTR similarities. We distinguished each of these eight groups as an HTR community (Fig. 3). Our enrichment analysis identified significant (*P* < 0.05) enriched community-specific infection attributes for each HTR community (Fig. 3 and Supplementary Table S4). Notably, Communities 1, 2, 3, 4, and 5 were enriched with cryptosporidium, RNA viruses, chronic/oncogenic infection pathogens, enveloped DNA viruses, and DNA tumor viruses, respectively. Meanwhile, Communities 6 and 7 were enriched with proteobacteria, whereas Community 8 was enriched with Picornaviridae and commensal bacteria.

Community-specific infection attributes overlapped for 14 (60.9%) of the 23 highly HTR-associated infection attributes, including enrichment of intracellular pathogens in Community 3 and enrichment of commensal pathogens in Community 8 (Supplementary Figure S7, Supplementary Tables S3 and S4). These results indicated that HTR similarities between pathogens within an HTR community were indeed related to certain highly HTR-associated infection attributes shared by these pathogens. The HTR community-delineated infection attributes counts exceeded those of the random divisions significantly (Supplementary Figure S8), suggesting that the HTR similarities of community component pathogens were robust and reliable.

**Figure 4:**
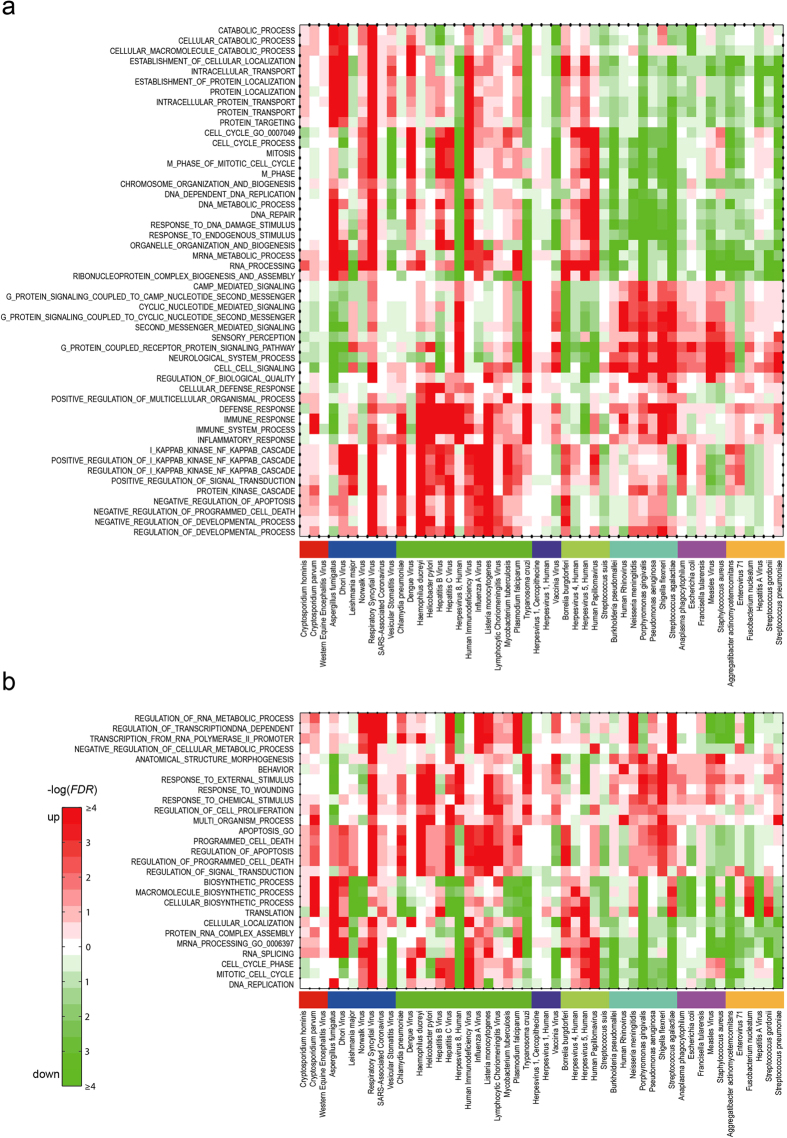
Community-specific and common bioprocesses. Heat map representation of (**a**) community-specific and (**b**) common GO BPs. GO BPs are colored according to calculated -log(*FDR*) values with red representing up regulation and green representing down-regulation. See Supplementary Table S5 for detailed information about community-enriched bioprocesses and Supplementary Data S6 for enrichment scores, *P* values, and *FDR* values.

To examine whether genes in a designated Gene Ontology Biological Process (GO BP) were consistently dysregulated to a significant extent in HTRs to pathogens within an HTR community, we calculated the enrichment scores of GO BPs in relation to pathogen mPRLs using GSEA (cutoff FDR < 0.01)^11^. We identified significantly dysregulated GO BPs in the HTRs to each pathogen as well as enriched GO BPs for particular HTR communities (Supplementary Data S6). In total, we identified 50 distinct community-specific GO BPs (Fig. 4a, Supplementary Table S5), some of which were functionallay related to corresponding infections. For example, catabolic process and intracellular protein transport are specifically activated in HTR Community 2, whereas mRNA metabolic process and intracellular transport are specifically inhibited in HTR Community 8; the immune/defense response, positive regulation of I KappaB/NF-KappaB cascade, and negative regulation of apoptosis/programmed cell death are highly activated in HTRs to oncogenic pathogens in Community 3^17^,^18^, but not in HTRs to the DNA tumor viruses in Community 5, in which G protein-coupled receptor signaling and sensory perception are specifically down regulated^19^,^20^. Notably, the same G protein-coupled receptor signaling is specifically activated in both HTR Communities 6 and 7^21^. However, pathogens in Community 6 cause additional diverse HTR dysregualtions, including specifically up regulated cell signaling and second messenger mediated signaling, as well as down regulated DNA metabolic process and repair, cell cycle (including M phase, mitosis), and response to DNA damage and endogenous stimulus. To some extent, these gene functional features helped distinguish proteobateria in Community 6 to those in Community 7. Together with annotated infection attributes, the gene expression pattern analysis validated our within-HTR community pathogen associations, further demonstrating the host response patterns to infections of different pathogen types are limited and differential.

Meanwhile, several GO BPs showed overlaps in dysregulated HTRs to multiple pathogen types (Fig. 4b), though not enriched in any particular HTR community. For example, genes involved in apoptosis are significantly up regulated in HTRs to pathogens in communities 2 (enriched of Mononegavirales) and 6 (enriched of respiratory flora bacteria), the pathogenicity of which have been reported to be highly related to this particular bioprocess^22^,^23^; genes involved in cell cycles are significantly down regulated in HTRs to pathogens in communities 6, 7, and 8 (enriched of proteobacteria and oral commensal bacteria, respectively), the effectors of which have been observed to inhibit proliferation and cause atrophy of epithelial cells^24^,^25^ These consistently dysregulated processes may be common HTRs^4^ that may facilitate our understanding of associations among pathogens in distinct case types.

### Associations among HTR community pathogens

Based on the categorization of 50 pathogens as HTR community components, we searched our annotated HTR community for: (i) genetically related pathogens with significant HTR similarity, (ii) genetically related pathogens with distinct HTRs, (iii) genetically unrelated pathogens with significant HTR similarities and underlying infection attributes/mechanisms, and (iv) HTR features common/specific to genetically unrelated pathogens with similar clinical manifestations. We confirmed the reliability, accuracy, and sensitivity of identifying external pathogens sharing the same HTR features upon querying HTR community reference profiles.

#### Proteobacteria

Notably, 10 of the 21 bacterial pathogens in our HTR Connectivity Map are proteobacteria, which is a taxonomic class composed of a variety of Gram-negative (i.e., with outer membrane) pathogenic genuses. These 10 proteobacteria species presented with significant HTR similarities (Supplementary Figure S7 and Supplementary Table S3), and all 10 are in HTR Community 6 or 7, with this classification pattern representing an enriched infection attribute (Supplementary Table S4).

To determine whether query signatures of HTRs to external proteobacteria (rough and simple gene sets) could be identified through HTR comparisons, we collected gene-expression profiles of cultured cells infected with wild-type and mutant *Salmonella enterica* subspecies *typhimurium* (Supplementary Signatures S1–5 and Supplementary Table S6), and analyzed *in vivo* gene-expression responses to *Burkholderia cepacia* infection (Supplementary Signature S6 and Supplementary Table S6).

**Figure 5:**
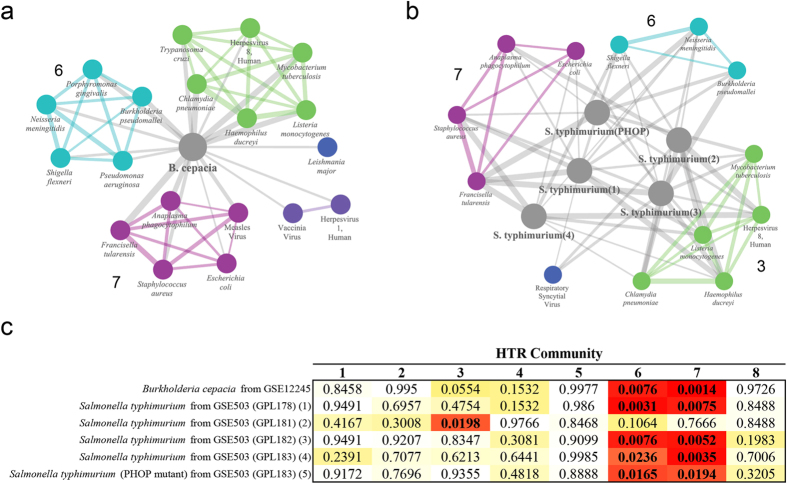
Proteobacteria HTR associations. Subcommunities connected to the external proteobacteria (**a**) *B. cepacia* and (**b**) *S. typhimurium* when each was integrated in the HTR Community. **(c**) Permutation *P* values describing the significance of associations between external pathogen signatures and each of the eight HTR communities. Grey magnified nodes represent query signatures generated from host transcriptional expression profiles (detailed information in Supplementary Table S6). For clarity, only pathogen pairs with association scores > 0.5 are shown. Permutation *P* values < 0.05 are shown in bold. Community colors are consistent with Fig. 3 and edge thickness is likewise proportional to association score. For clarity, edges were connected for *Salmonella typhimurium* and internal pathogens only if the HTR community yielded a positive association score > 0.5 for at least 3 of 5 query signatures. HTR associations among the 10 internal proteobacteria in the HTR Connectivity Map are shown in Supplementary Figure S9 and reported in Supplementary Data S7. Association scores between external query signatures and mPRLs are reported in Supplementary Data S8.

Upon querying, we observed marked positive associations of *B. cepacia* with HTR Communities 6 and 7 (Fig. 5a,c). Analogous results were seen for four out of the five *S. typhimurium* query signatures generated on four different microarray platforms (Fig. 5b,c). One *S. typhimurium* query signature (derived from an experiment using a *phoP::Tn10* mutant strain that replicates intracellularly but is defective for killing cultured and primary human macrophages) was also associated strongly with HTR community 6 (Fig. 5b,c), demonstrating the high sensitivity of HTR Community analysis for identifying characteristic HTRs to a specific pathogen class. As a whole, these results indicate that a query signature derived from a class of microorganisms with consensus HTRs can be used to pull up other taxonomically aligned microorganisms.

#### Streptococcus

The four spherical Gram-positive *Streptococcus* species in the HTR Connectivity Map—*S. gordonii*, *S. pneumonia*, *S. suis*, and *S. agalactiae*—have varied pathogenicity related to their differing hemolytic properties^26^. *S. agalactiae* is a beta-hemolytic species that causes complete hemolysis, whereas the three others are alpha-hemolytic species that cause partial hemolysis. Herein, we tried to evaluate the ability of annotated HTR Community analysis to differentiate the pathogenicity of internal and external *Streptococcus* species.

**Figure 6:**
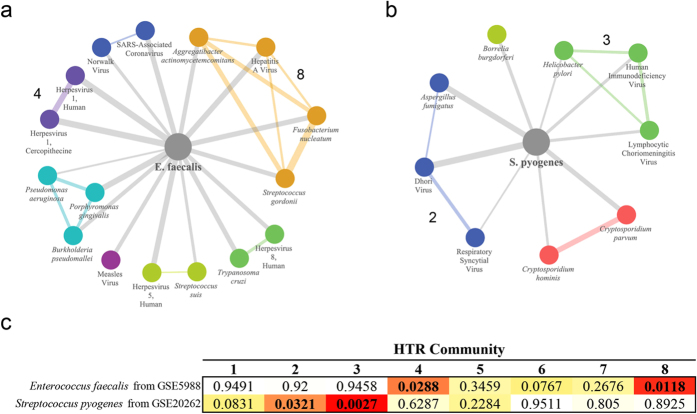
*Streptococcus* HTR associations. Subcommunities connected to the external (formerly) streptococcal bacteria (**a**) *E. faecalis* and (**b**) *S. pyogenes*, when each was integrated in the HTR community. (**c**) Permutation *P* values for associations between external pathogen signatures and each HTR community. Grey magnified nodes represent the query signatures generated from host transcriptional expression profiles (detailed information in Supplementary Table S6). For clarity, only pathogen pairs whose association scores were > 0.5 are shown, and permutation *P* values < 0.05 are bolded. Color scheme is consistent with Figs 3 and 5, and edge thickness is proportional to association score. Association scores between external query signatures and mPRLs are reported in Supplementary Data S8.

The internal *Streptococcus* species *S. gordonii*, an oral commensal bacterium, and *S. pneumonia*, carried asymptomatically in the nasopharynx, can be pathogenic in susceptible individuals^27^. Accordingly, they were both classified into HTR Community 8 (Fig. 3), which is enriched with alpha-hemolytic and commensal bacteria. Formerly classified as part of the Group D *Streptococcus* system, *Enterococcus faecalis* is an alpha-hemolytic commensal inhabitant in the human gastrointestinal tract^28^. When we queried our HTR Community dataset with collected signatures from gene expression profiles generated from *E. faecali*-infected human urothelial cells (Supplementary Signatures S7 and Supplementary Table S6), we confirmed that indeed this external *Streptococcus* specie also showed strongest positive associations with HTR Community 8 (Fig. 6a,c).

Beta-hemolytic *Streptococcus* species are subdivided into 20 serotypes (Lancefield groups A to V) describing their cell-wall carbohydrates, with Lancefield groups A and B being the most clinically important groups^26^. The internal species *S. agalactiae*, also known as Group B streptococcus (GBS), is an opportunistic pathogen of the normal gut and genital tract flora, with a polysaccharide antiphagocytic capsule being its main virulence factor^29^. Consequently, it was classified in HTR Community 6 (Fig. 3), which is enriched with encapsulated human flora bacteria.

*S. pyogenes*, an external *Streptococcus* also known as Group A streptococcus (GAS), causes many diseases, ranging from mild superficial skin infections to life-threatening systemic diseases^30^. It also causes post infectious non-pyogenic syndromes, including rheumatic fever and acute post infectious glomerulonephritis^31^. Its pathogenicity is associated with several GASs common (e.g., Streptolysin O and S) and specific (e.g., *Streptococcal pyrogenic* exotoxin A and C^32^, and Streptococcal chemokine protease^33^) virulence factors that enable the bacterium to attach to host tissues, evade immune responses, and spread by penetrating into tissue layers.

Given its distinct characteristics, we hypothesized that *S. pyogenes* would not co-segregate with *S. agalactiae* in HTR Community 6. Thus, we collected the only available gene expression profiles generated from samples of blood, saliva, and throat swabs from *S. agalactiae*-infected Cynomolgus macaques (Supplementary Signatures S8 and Table S6), queried the HTR Communities, and indeed found that *S. pyogenes* associated most strongly with Community 3 (permutation *P* = 0.0027, Fig. 6b,c), in which immune-related BPs are significantly activated. Collectively, these results show that HTR community analysis can identify distinguishable associations among phylogenetically related pathogens with differential underlying pathogenicity, at least in the present sample.

#### Oral commensal bacteria

To further test the capacity of the HTR community method for identifying distinct mode of functional associations among pathogens, we then explored genetically unrelated bacterial pathogens with significant HTR similarities.

Oral commensal bacteria are highly diverse and inhabit the various surfaces of the mouth. Their ability to form biofilms on hard and soft oral tissues makes them important in periodontal disease^34^. Our 50-pathogen HTR Connectivity Map includes one oral commensal species *S. gordonii* and two opportunistic oral commensal species, *Aggregatibacter actinomycetemcomitans* and *Fusobacterium nucleatum*. Intriguingly, these three species showed significant HTR similarities (Fig. 3). Of note, the oral commensal bacterium characteristic is also an enriched infection attribute for bacteria in HTR Community 8.

Surprisingly however, *Porphyromonas gingivalis*, a periodontal pathogen found in the mouth, upper gastrointestinal tract, respiratory tract, and colon, did not positively associate with these three oral (opportunistic) commensal bacteria, but rather was classified into HTR Community 6, which is enriched with pathogenic respiratory flora and Gram-negative rod bacteria (Fig. 3). Further evidence from gene expression pattern analysis showed that *P. gingivalis* shared the GO BP of activated G protein-coupled receptor pathway with Community 6, a feature not enriched in Community 8 (Fig. 4a).

**Figure 7:**
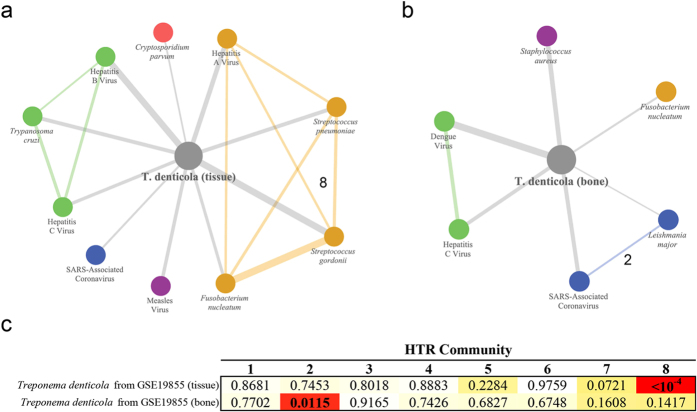
Oral commensal bacterium HTR associations. Subcommunities connected to the external bacterium *T. denticola*, whose infection HTR was derived from **(a)** soft tissues overlying calvarial bones and **(b)** calvarial bones when *T. denticola* was integrated in each HTR community. **(c)** Permutation *P* values for associations between external pathogen signatures and each HTR community. Grey magnified nodes represent query signatures generated from host transcriptional expression profiles (detailed information is provided in Supplementary Table S6). For clarity, we included only pathogen pairs with association scores > 0.5, and permutation *P*-values < 0.05 are shown in bold. Edge thicknesses are proportional to association scores; edge and node colors are consistent with Figs 3, 5 and 6. Association scores between the external query signatures and the mPRLs are reported in Supplementary Data S8.

To validate the specificity of this highly oral microbiota related HTR, we selected another oral commensal bacterium, *Treponema denticola,* with which to query the HTR Community. The only available *T. denticola* query signature was generated from a report documenting differentially expressed genes in a murine model of *T. denticola* head infection (Supplementary Signatures S9 and S10; Supplementary Table S6). The conditions used in that study differed sharply from those used to build the HTR Connectivity Map with respect to RNA source (calvarial bones and overlying soft tissues *vs*. cell lines) and species (mouse *vs*. human). Nonetheless, HTR community analysis yielded the strongest positive association with HTR Community 8 for the *T. denticola* query signature derived from calvarial overlying soft tissues, but not those from calvarial bones (Fig. 7b,c). This dissociation is likely due to the fact that the three reference profiles in the HTR Connectivity Map were not derived from calvarial bones (Supplementary Table S1), whose expressed transcripts are generally not shared with other cell or tissue types. These findings demonstrated again that human microbiota with distinguishable host gene expression patterns can be identified by HTR community analysis.

#### Respiratory viruses

Finally, we sought to use the HTR community method to generate hypotheses about a unique HTR characteristic within a group of pathogens whose infections have indistinguishable local and systemic manifestations, but differing prognoses. For this purpose, the respiratory viruses fall in our focus.

HTR Connectivity Map included five respiratory viruses from four distinct families, namely influenza A virus (IAV) and Dhori virus in the family *Orthomyxoviridae*, human respiratory syncytial virus (RSV) in the family *Paramyxoviridae*, Severe Acute Respiratory Syndrome-associated coronavirus (SARS-CoV) in the family *Coronaviridae*, and human rhinovirus (HRV) in the family *Picornaviridae*. All five viruses are associated with high morbidity and their infections cause similar minor (e.g., coughing, sore throat, runny nose, and fever) and severe symptoms (e.g., severe breathing problems, bronchiolitis, bronchitis, and pneumonia) in humans^35^,^36^. To our surprise, these five viruses were classified into three different HTR Communities (Fig. 3): HRV was classified into HTR Community 6 (enriched with pathogenic respiratory flora), IAV into Community 3 (enriched with pathogens with oncogenic potential after chronic/persistent infection), and Dhori virus, SARS-CoV, and RSV into Community 2 (enriched with *Mononegavirales* order pathogens).

**Figure 8:**
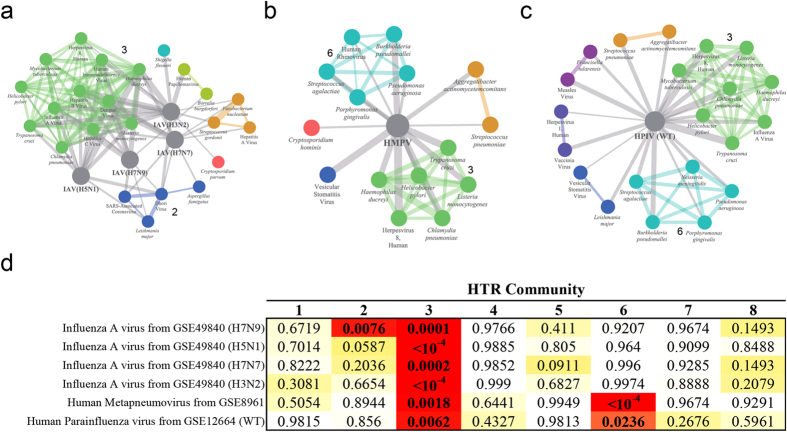
Respiratory virus HTR associations. Subcommunities connected to external respiratory viruses, including **(a)** IAV, **(b**) hMPV, and **(c)** hPIV (wild-type), when each was integrated into the HTR community. **(d)** Permutation *P* values for associations between external pathogen signatures and each HTR community. Grey magnified nodes represent query signatures generated from host transcriptional expression profiles (detailed information is provided in Supplementary Table S6). For clarity, we included only pathogen pairs with association scores > 0.5, and HTR communities with a permutation *P* value < 0.05 are shown in bold. Edge thicknesses are proportional to association scores; edge and node colors are consistent with Figs 3 and 5, 6, 7. Association scores between external query signatures and mPRLs are reported in Supplementary Data S8.

To validate the unique HTR to IAV, we first employed four IAV query signatures from a recent report in which human lung epithelial cells were infected with a novel avian-origin H7N9 strain, two highly pathogenic avian-origin H5N1 and H7N7 strains, and a human seasonal H3N2 strain (Supplementary Signatures S11–14 and Supplementary Table S6). For all four external IAV strains, the HTR community analysis yielded consistently strong positive associations with Community 3 (permutation *P* < 0.001) (Fig. 8a,d).

We then proceeded to investigate the HTR associations of two other clinical important respiratory viruses in the *Paramyxoviridae* family that were not included in the HTR Connectivity Map, namely human metapneumovirus (hMPV) and human parainfluenza virus (hPIV). Both query signatures were generated from expression profiles of human lung epithelial cells after time-course infections (Supplementary Signatures S15 and S16; Supplementary Table S6). HTR community analysis yielded the strongest positive associations for hMPV and wild-type hPIV-1 with Community 6 (permutation *P* < 10^−4^ and =0.0062, respectively) (Fig. 8b,d), despite that they also showed positive associations with Community 3 (permutation *P* = 0.0236 and =0.0018, respectively) (Fig. 8c,d).

Altogether, these results indicated that respiratory infections with indistinguishable clinical manifestations may differ greatly in HTRs. This makes delicate HTR classification of individual infection type, especially the newly emerged viral strain(s), constantly needed to better understand the common and specific HTR features. Although in a preliminary stage, the findings about known respiratory viruses and especially IAV, which is uniquely responsible for the highly contagious influenza outbreaks, underscore the necessity for specific host-directed antiviral strategies in epidemic control.

## Discussion

Resource projects such as the Connectivity Map^10^ and the subsequent Library of Integrated Network-based Cellular Signatures^37^, which provide an expansive library of post-drug treatment gene expression profiles, are of high operability. Development of a similar platform cataloguing gene expression profiles characterizing HTRs to particular pathogenic infections is needed. The present work represents a pilot venture toward fulfilling that need.

An essential advantage of using transcriptional bioinformatics in drug discovery and repositioning lies in the fact that plentiful information—including chemical, pharmacological and pharmaceutical data, with comprehensive information about drug targeting (i.e. sequence, structure, and pathway)^38^ and adverse secondary effects^39^—has been curated for small-molecule drugs. This plentiful information has served as an excellent annotation resource and facilitated the elucidation of drug mechanisms, as well as the identification of new drug targets and new indications for old drugs^40^,^41^,^42^.

The HTR Connectivity Map scheme developed here was limited to publically accessible expression profiling data, therefore, our data collection was unavoidably at risk of bias. Recognizing the limited cell type diversity among our samples in our systematic assessment of HTR similarities, we combined PRLs of a specific pathogen computationally to represent the integral feature of HTRs to that pathogen. Through further clustering, we identified eight pathogen groups, that is, HTR Communities, with a discernible consensus of internal HTR similarities. The methodologies used in this study, including gene expression profile merging, comparison, and clustering, were first introduced by Iorio *et al.*^12^ and Subramanian *et al.*^11^ Previously, these methods performed very well in characterizing and predicting similarities in drug effect and mode of action across cell lines and dosages, and further in partitioning drugs into communities (i.e., compounds with similar modes of action). Moreover, it was our aim to complement this HTR Community as a resource and elucidate the HTR associations established among the 50 pathogens. To this end, we collected laboratory and clinical infection characteristics for individual pathogens, and then identified computationally HTR-related infection attributes, as well as differential gene expression patterns, for each HTR community. These annotations greatly facilitated the understanding of correlations between pathogen types and significant HTR similarities, as well as the underlying infection mechanisms.

Overall, the associations established in our pilot 50-pathogen HTR Community are biologically revealing. We demonstrated that the HTR landscape of pathogenic infections is complex but composed of delimited and differential patterns. Using four cases, we illustrated that such resource and analysis provide for the first time: (1) the correspondence of pathogen taxonomy with HTR classifications, which makes external/new pathogen(s) and specific infection feature(s) identifiable upon signature querying of the reference profiles in HTR Communities (e.g., the proteobacteria and oral commensal bacteria cases); (2) the common and specific HTR community gene expression patterns, which empower the elucidation on shared and distinct molecular mechanisms of host cells in confrontation with individual pathogen types (e.g., the *Streptococcus* case); (3) HTR categorization and differentiation of clinically related pathogens, which generate new biological hypotheses, and inform experimental validation and host-directed anti-infection therapies (e.g., the respiratory viruses case). Importantly, we employed as many query signatures of HTRs to external pathogens as possible to challenge the proposed associations, and the positive results provided strong evidence confirming the robustness of the HTR community constitution and the reliability of our findings.

Nevertheless, due to limitations in pathogen type coverage, it was still challenging to accurately categorize HTRs to taxonomical class with our first-generation HTR community. For example, in the *Streptococcus* case, the internal species *S. Suis* was classified into HTR Community 5 rather than Community 8, where other alpha-hemolyte bacterial species reside (Fig. 3). This dissociation is likely related to the fact that *S. Suis* is primarily a commensal and opportunistic swine/pig pathogen, with human infections being infrequent but grave when an outbreak does occur^43^. Besides, the enriched GO BPs obtained for HTRs to *S. Suis* were divergent from those of the other four pathogens in HTR Community 5, which are DNA tumor viruses (Fig. 3).

Also in the proteobacteria case, the internal protebacteria *A. actinomycetemcomitans* showed negative associations with two of the nine other proteobacteria, i.e., *Helicobacter pylori* and *Neisseria meningitidis* (Supplementary Figure S9 and Supplementary Data S7). Besides, it was categorized in HTR Community 8 as an oral commensal bacterium, rather than as a member of HTR Community 6 or 7, where the other examined proteobacteria were classified. We then found by the gene expression pattern analysis that HTR to *A. actinomycetemcomitans* infection was unusual among proteobacteria in that it did not include up regulated G protein coupled receptor protein signaling (Fig. 4a). This highly indicated that for pathogen type assigned with multiple enriched attributes, combined results from gene expression pattern analysis should be used to elucidate the essential and differential characteristics of host-pathogen interaction.

Another problem in full discovering HTR associations using the first-generation HTR Community lies in the fact that the host cell type diversity is limited, i.e., mostly blood cells, epithelial cells, and cancer cell lines. It compromises the ability of HTR community, as a resource of reference profiles, to find reliable associations with other diverse cell types (e.g., bone cells). This was exactly the case for querying *T. denticola* with internal oral commensal bacteria, in which we failed to observe positive associations for query signatures derived from calvarial bones (Fig. 7c). As suggested in cMap^10^, this particular event reminds again that, to maximize HTR community sensitivity in signature-based discovery of functional associations, reference profiles should be collected in as many cells/tissues as possible to assure appropriate, systemic exhibition of normal and extreme physiological contexts.

In addition, interpretation of HTR community results depends on the ability to identify associations with higher confidence, including deciphering the meaning of dual associations. In the respiratory viruses case, dual associations were observed for query signatures of hMPV and wild-type hPIV-1, raising questions about the reliability of pathogen-to-community associations. However, we learned from the literature that—similar to IAV—hPIV and hMPV also have hemagglutinin-neuraminidase and functionally similar proteins (e.g., fusion protein F) on their surfaces that serve as antigenic and virulent markers^44^,^45^. Moreover, their dual community associations can be explained by the shared gene expression patterns in HTR communities 3 and 6. Specifically, HTRs to pathogens in Community 3 showed enrichment in positive regulation of NF-kappaB signaling and immune responses, as well as negative regulation of apoptosis, whereas HTRs to pathogens in Community 6 showed enrichment in decreasing host cell mitotic activity (Fig. 4). Consistent with these findings, NF-kappaB signaling is induced strongly by hMPV and hPIV infection^46^,^47^,^48^ and decreased cell mitotic activity has been reported to occur following hPIV infection^49^. These findings highlight the necessity to increase the specificity of annotated HTR community enriched infection attributes and gene-expression patterns.

On the basis of the results of this pilot study, we propose that a sensible next step would be the generation of an expanded HTR Connectivity Map to be used as a public resource. Additional reference profiles incorporating a broader taxonomic representation of pathogens and cell-type diversity, together with *in vivo* data, should be incorporated into the expandable HTR Connectivity Map to improve community characterization and feature identification. More rigorous methods for determining statistical significance should improve annotation trustworthiness and strengthen the reliability of inter-pathogen HTR associations, especially as the size of the reference profile database grows.

A larger scale HTR community resource will enable HTRs to diverse pathogens to be analyzed with higher accuracy, sensitivity, and reliability. Moreover, researchers studying HTRs to an individual pathogenic species or a group of genetically-related or clinically-associated pathogens could compare target species signatures to reference profiles, This could lead to unexpected connections and biological hypotheses for in-depth experimental validations. Ultimately, the advanced HTR Connectivity Map will improve our understanding of pathogens of interest when their community affiliations are defined, and with the addition of further experimental evidence, propel discovery of molecular mechanisms mediated by multiple cell types in a coordinated response to infections, as well as the development of host-directed antimicrobials.

## Methods

### Reference profile collection

Resources containing expression profiles of host cellular responses to pathogenic infections were collected by manual searching and expert reviewing of dataset descriptions in the Gene Expression Omnibus database. To limit our analysis to genome-wide gene expression changes, the gene expression profile data were produced with only Affymetrix Human Genome U133A Array and U133 Plus 2.0 Array platforms. Our criteria for project inclusion were threefold: (1) at least one sample of untreated specific pathogen infection with infectious disease state or *in vitro* infection for at least 1 h; (2) at least one control sample (e.g. uninfected, mock-infected, healthy control or other blank control); (3) data processing methodology clearly defined in series matrix file, data values in series matrix file distributed in a regular fashion (e.g., log-scale distribution for count values processed with MAS5; approximate normal distribution for log-transferred values processed with RMA), and no more than 1% of data values missing. And the original data collected were provided in our lab website(http://biotech.bmi.ac.cn/papers/2015/luhan.html).

### Generating PRLs

Each pair of samples containing one infection sample and one corresponding control sample was considered an instance. We paired infection samples and control samples in accordance with five principles:

1. Very early infection sample measurements (<1 h) were not taken.
2. Pathogen infection samples were paired with control samples such that experiment conditions (i.e. cell type and culture time) were identical.
3. Samples measured before infection or at infection time 0 were treated as controls if there were no control samples measured after time 0.
4. If the number of control samples exceeded the number of infection samples, the excess control samples were omitted.
5. If the number of infection samples exceeded the number of control samples, the excess infection samples were omitted unless they were designated as simple repeated measurements or replications of the same experimental condition. In such cases, the excess infection samples were paired with used control samples in a revolving fashion. For example, if there were two control samples c1 and c2 and five infection samples i1 to i5, then i1, i3, and i5 would be paired with c1, while i2 and i4 were paired with c2.

The intersection of probes for each dataset was generated to obtain the final probes shared by all datasets. Sample data values from series matrix files were transformed into count values if they had been log transformed. The probes were ranked according to the expression change produced by comparing corresponding infection and control samples. First, sub threshold instance values were set to a threshold value. The 25^th^ percentile level of the instance was selected as the corresponding threshold value. Next, probe sets were ranked in descending order of the corresponding perturbation-to-control value ratios. For probe sets with a ratio that equaled one, a lower threshold (the 25^th^ percentile divided by 10) was applied. Finally, the probe sets were subsorted in descending order of the new ratio calculated. The sorted probe lists constituted PRLs and represented regulation level that considered both fold-changes in expression and expression values. The probes with the most up- (or down-) regulated genes had top (or bottom) PRL rankings. A total of 893 PRLs, denoting infection by 50 pathogens across different cell lines and from different laboratories, were obtained.

### Merging PRLs for an individual pathogen

Spearman’s Foot rule was used to measure inter-PRL distances. For given PRLs A and B, the ranking of probe identifiers *P*_*1*_, *P*_*2*_, *P*_*3*_,…, *P*_*m*_ (*m* = 22, 160 in our study) in PRL A and B are represented by *A*_*1*_, *A*_*2*_, *A*_*3*_,…, *A*_*m*_ and *B*_*1*_, *B*_*2*_, *B*_*3*_,…, *B*_*m*_, respectively. Spearman’s Foot rule correlations between PRL A and B were computed with the following formula:


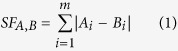


Spearman correlation coefficients were calculated between each pair of PRLs (Supplementary Figure S1).

An iterative process was applied to merge PRLs of the same pathogen by building a minimum spanning tree in accordance with the Kruskal algorithm strategy^50^ and merging the PRLs of each pathogen with a minimum spanning tree in accordance with the Borda merging method^12^,^13^,^51^. In the iterative process, the two PRLs with the closest Spearman’s footrule values were combined and replaced by a single PRL. This iterative process was repeated until only one mPRL remained.

The Borda merging method is a consensus-based voting algorithm. For PRLs A and B, the ranking of all probe identifiers (*P*_*1*_, *P*_*2*_, *P*_*3*_,…, *P*_*m*_) would be *A*_*1*_, *A*_*2*_, *A*_*3*_,…, *A*_*m*_ and *B*_*1*_, *B*_*2*_, *B*_*3*_,…, *B*_*m*_, respectively. The ranking weight of *P*_*i*_ was defined as follows:


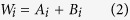


A new ranked list, the mPRL of all probe identifiers, was obtained by sorting *W*_*1*_, *W*_*2*_, *W*_*3*_,…, *W*_*m*_ in increasing order.

PRLs for the same pathogen, across different host cell types and from different labs, were combined into a single PRL in R package GeneExpressionSignature software^22^ such that the HTRs of each individual pathogen were combined according to a hierarchical majority-voting scheme as described previously^15^,^22^. A single synthetic mPRL was computed by merging all the PRLs referring to the same pathogen, such that genes that were consistently up- or down-regulated across individual PRLs were placed at the top or bottom, respectively, of the mPRL. PRLs of different strains or subtypes of the same pathogen species were computationally merged if their expression profiles from centralized projects were similar to each other. For example, the PRLs of several subtypes of oncogenic human papillomavirus (HPV), including HPV-16, HPV-18, HPV-31, HPV-33, HPV-35, HPV-58, HPV-66, were merged for the HPV infection HTR profile.

We calculated Spearman correlation coefficients between the mPRL and individual component PRLs to see if the mPRL captured the infection features of the component PRLs. Theoretically, an mPRL for a specific pathogen should correlate strongly with the component PRLs for the same pathogen, and relatively more weakly with component PRLs for other pathogens. That is, in an ROC depicted with individual component PRLs as benchmarks, the AUC should approach 1. We found that the AUCs for 38/50 component pathogens were 1, and 45/50 were >0.9, with an average AUC of >0.97 for all 50 pathogens (Supplementary Figure S5).

### Calculating HTR similarities across pathogen pairs

We represented pathogen-to-pathogen HTR relationships as association scores computed with a GSEA-based PRL comparing method^10^,^11^,^14^. A signature was extracted for each pathogen, where a signature refers to a group of genes that may serve as a synthetic descriptor of a particular biological action (e.g., a disease, cellular drug response, etc.). In our study, each signature was a subset of the most consistently differentially regulated genes in the general cellular responses to pathogen infections.

We selected the highest- and lowest-ranked 250 genes from each PRL as a pathogen signature. The GSEA-based PRL comparing method is a parameter-free algorithm, with the exception of signature size (recommended range, 15–500 per gene set, with lower size increasing randomness and large size decreasing specificity). We estimated the size parameter influence by sampling from 50 to 450 in intervals of 50. Pearson correlation coefficients of the association scores between 1,225 pairs of HTR relations indicated that the association scores obtained within this tested signature size range correlated with one another robustly (Supplementary Figure S6). The strongest correlation (mean coefficient >0.97) was obtained with a signature size of 250 genes (Supplementary Figure S6), demonstrating a limited influence of signature size. Therefore, we used a signature size of 250 for further analyses.

To evaluate HTR similarities across different pathogens, we used the GSEA^11^ method, which is based on the Kolmogorov-Smirnov statistic, to quantify whether signature genes tend to have similar ranks in the PRLs of two compared pathogens (i.e. top or bottom) and presented the outcome as an enrichment score. We used {up_A_, down_A_} to represent the signature of pathogen A, and the enrichment score of up_A_ (or down_A_) in the PRL for pathogen B, which was represented by 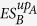 (or 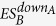) and would be high if the corresponding genes tended to be placed at the top (or bottom) of the PRL for pathogen B. If the length of PRL is *m*, and the {up_A_} contains *n* probe identifiers ranked *R*_*1*_, *R*_*2*_, *R*_*3*_,…, *R*_*n*_ in PRL B, then 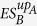 could be obtained as follows:


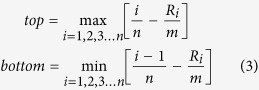


The association score was *top* when 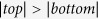 or *bottom* otherwise.

HTR similarity between pathogens A and B was expressed by an association score between them drawn from the enrichment scores of their signatures in the opposing pathogen’s PRL. We defined the association score between HTRs of pathogen A and B as follows:


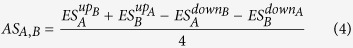


To validate the significance of the association scores, we used the same algorithm to calculate the association score between two random PRLs of the same size as those used in our study (i.e., 22, 160) to obtain a control. We repeated this experiment one million times. We computed a *P* value for each pairwise pathogen association score by comparing the actual values to the distribution of values obtained for the random data comparison. The *P* value was estimated as the frequency that random control values exceeded the actual value. *FDR* values were estimated as described by Benjamini and Hochberg^52^.

### Identification of HTR-associated pathogenic infection attributes

Pathogenic infection attributes were collected according to four major categories: (1) biological pathogen classification of MeSH; (2) tissues/cells affected by the infection according to MeSH and the literature; (3) infectious diseases or symptoms according to MeSH and the literature; (4) Other important clinical (e.g., staining, intracellular/extracellular, shape, capsulation, respiration, motility, envelopment, replication site) and laboratory characteristics (e.g., transmission and disease manifestation) of pathogenic infections, as represented in key words.

The infection attribute terms were hierarchical descriptors for the studied pathogens. The association scores between each pair of pathogens were sorted in descending order. We selected pathogen pairs that shared the same descriptors and recorded their rankings. Supposing there are *N* pairs of pathogens, and *n* pairs of pathogens sharing the same descriptor X, the rankings of these pathogen pairs sharing the same descriptors were represented as *R*_1_*, R*_2_*, R*_3_ *… R*_*n*_. We used the Kolmogorov–Smirnov statistic to generate an association score for each descriptor that represented the level of HTR similarity between the paired pathogens. The association score for each descriptor was generated by computing the following values:


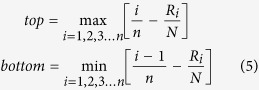


The association score was *top* when 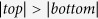 or *bottom* otherwise.

One million (*M*) random permutations of the pathogen pairs were generated to estimate the permutation *P* value of each character term. For *M* random permutations, the quantity of association scores obtained was *m*; if the random permutation association score was not less than the actual association score of that character, the frequency of this event (*m/M*) was taken as the permutation *P* value. To improve association test reliability, only character terms shared by at least five pathogens were tested. *FDR* values were again estimated as described by Benjamini and Hochberg^52^. Character terms with an *FDR* value <0.01 were accepted as characteristics significantly related to HTR.

### Identifying HTR communities and component analysis

The parameter-free affinity propagation algorithm^16^ was used to identify pathogen clusters, that is, communities with significantly similar internal HTRs. With a hypergeometric distribution, the enrichment analysis results of infection attributes in each community were expressed as *P* values, representing the probability that an infection attribute occurrence number exceeds its actual number. When a total of *N* pathogens are clustered into several communities, and the target community is of size *n*, and *m* of *N* pathogens share the same infection attribute X, and *k* of them are assigned to the target community, then the *P* value used to check whether target infection attributes X is enriched in target community can be expressed as follows:


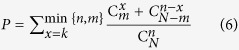


### Identifying community-common and -specific bioprocesses

GO BP signatures were downloaded from the Molecular Signature Database on May 18, 2015. Bioprocesses with a signature size in the range of 50–500 were selected to serve as a reference database, where fewer genes increases randomness and more genes reduces specificity. The enrichment score of each gene signature in each pathogen PRL was generated by GSEA. Supposing a PRL length of *m* and that a bioprocess signature contains genes corresponding to *n* probe identifiers, with the corresponding probe identifiers ranked *R*_*1*_, *R*_*2*_, *R*_*3*_,…, *R*_*n*_ in each pathogen’s PRL, then the enrichment score of the bioprocess signature in the PRL was computed as follows:


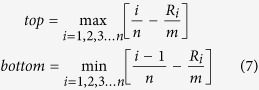


The enrichment score was *top* when 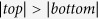 or *bottom* otherwise.

Ten thousand (*M*) trials (the corresponding values for random permutations of probe identifiers in each PRL) were used to estimate permutation *P* values for each enrichment score. The enrichment score of a bioprocess signature in a pathogen PRL was represented by *ES*, the corresponding trials by *ES*_*i*_ (*i* = 1, 2, 3…*M*), and the number of instances with 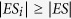 as *m*. The frequency (*m*/*M*) was taken as a two-sided *P* value. *FDR* values were estimated as above^52^. Bioprocess-pathogen relations with an FDR <0.01 were designated as significant.

A positive or negative ES indicated that the bioprocess was significantly activated or inhibited, respectively, during pathogen infection. Assuming a hypergeometric distribution, the enrichment analysis results of significantly activated or inhibited bioprocesses for each community were expressed as *P* values. If the total number of pathogens is *N*, and n of them are clustered in a community, and a bioprocess was considered to be significantly dysregulated in same direction in the infection of *m* of *N* pathogens, and *k* of them were in the target community, then the *P* value used to check whether the bioprocess was enriched in the target HTR community can be expressed as follows:


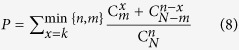


The threshold for designation of a bioprocess being enriched within a community was *P* < 0.01. Bioprocesses were considered common if they were dysregulated in the mPRLs of at least 10 pathogens.

### Generating query signatures of HTRs to external pathogenic infection from publicly available gene expression profiles

We used the R-based web application GEO2R, which enables users to identify differentially expressed gene sets within individual samples in a Gene Expression Omnibus dataset^53^. Each pathogen’s signature consisted of the 500 most significantly regulated probes identified by GEO2R. The hPIV infection data were provided in fold-change form, rather than expression values; therefore, the hPIV PRLs were combined by the Borda merging method^51^, and genes correlating with the top and bottom 250 probes were selected to represent the signature.

### Comparing query HTR signatures to those of external pathogenic infection with 50 pathogen reference profiles

GSEA was used to generate the enrichment scores for up- and down-regulated genes in the query signatures for each pathogen’s PRL. Supposing the enrichment scores for corresponding gene sets are designated as *ES*_*up*_ and *ES*_*down*_, the enrichment score of the pathogen signature can be expressed as *ES* = (*ES*_*up*_*−ES*_*down*_)/2. For a total of *N* pathogens, where *ES*_*i*_ is the enrichment score of pathogen *i*’s signature, 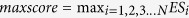, and 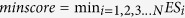, the association score of the signature for pathogen *i* was 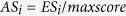 if 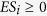 or 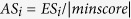 if 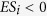.

### Measuring associations between query signatures of HTRs to external pathogenic infection and HTR communities

The 50 pathogens used to produce our HTR community scheme were ranked by association score relative to a query signature in descending order. Supposing a community contains *n* of a total *N* pathogens, and their rankings are *R*_1_, *R*_2_, *R*_3_ … *R*_*n*_, the association score between a gene signature and a community based on a Kolmogorov-Smirnov statistic was obtained as follows:


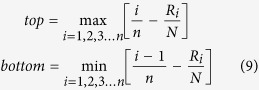


The association score was *top* when 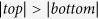 or *bottom* otherwise.

We used one million (*M*) random trails, as above, to estimate the permutation *P* values for each association. Supposing the association score between a gene signature and a community is *AS*, the corresponding trials were *AS*_*i*_ (*i* = 1, 2, 3…*M*). The number of instances with
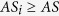
was counted as *m*, and the frequency (*m*/*M*) was taken as the *P* value.

## Additional Information

**How to cite this article**: Han, L. *et al.* Inferring Infection Patterns Based on a Connectivity Map of Host Transcriptional Responses. *Sci. Rep.* **5**, 15820; doi: 10.1038/srep15820 (2015).

## Supplementary Material

Supplementary Information

Supplementary Signature S1

Supplementary Signature S2

Supplementary Signature S3

Supplementary Signature S4

Supplementary Signature S5

Supplementary Signature S6

Supplementary Signature S7

Supplementary Signature S8

Supplementary Signature S9

Supplementary Signature S10

Supplementary Signature S11

Supplementary Signature S12

Supplementary Signature S13

Supplementary Signature S14

Supplementary Signature S15

Supplementary Signature S16

Supplementary Data S1

Supplementary Data S2

Supplementary Data S3

Supplementary Data S4

Supplementary Data S5

Supplementary Data S6

Supplementary Data S7

Supplementary Data S8

## Figures and Tables

**Figure 1:**
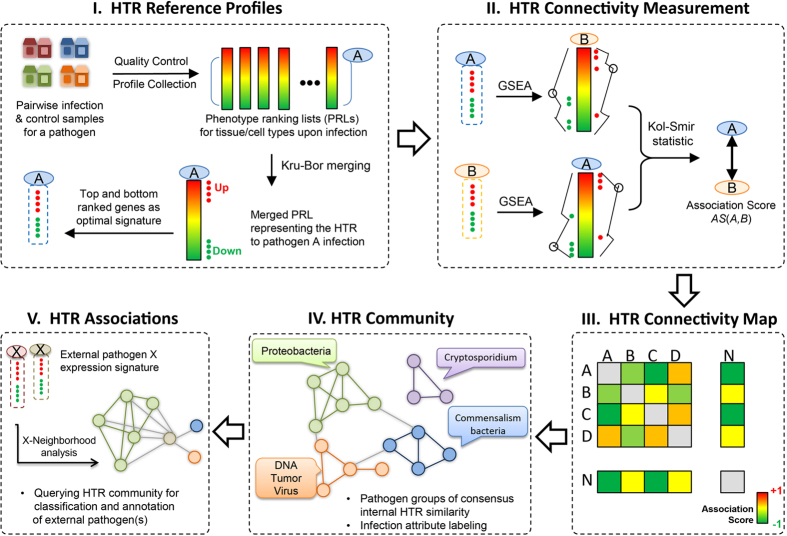
HTR Connectivity Map development work flow. A group of 1,353 expression profiles generated from infection of cultured human cells with 50 clinically important pathogens were collected from Gene Expression Omnibus and used to populate a reference database. A single synthetic PRL (22,160 genes ranked according to their differential expression relative to the control) was computed to represent consensus and common HTRs to infection with a pathogen across different cell lines and from different laboratories. GSEA was used to score each reference profile for the direction and strength of enrichment with the query reference signature. The connectivity of HTRs between pathogen pairs was then presented in a map/matrix, with positive scores indicating functionally similar HTRs and negative scores indicating opposing HTRs. Through clustering, 50 pathogens were grouped into eight HTR communities (i.e., pathogens that induce more consensus HTRs). Characteristic infection attributes and gene expression patterns were identified for each HTR community. HTR community reliability for demonstrating associations among pathogens was tested with query signatures of HTRs to external pathogens.
