# Supplementary material for: Inferring Infection Patterns Based on a Connectivity Map of Host Transcriptional Responses
Source: Sci Rep. 2015 Oct 28;5:15820. doi: 10.1038/srep15820 (PMC4623713; doi:10.1038/srep15820)
Supplement: Supplementary Information [file srep15820-s1.pdf]

Inferring Infection Patterns Based on a Connectivity Map of Host Transcriptional Responses

Lu Han[1,2], Haochen He[1], Fei Li[1], Xiuliang Cui[1,3], Dafei Xie[1], Yang Liu[1], Xiaofei Zheng[4], Hui Bai[1,5*], Shengqi Wang[1*], Xiaochen Bo[1*]

[1]Department of Biotechnology, Beijing Institute of Radiation Medicine, Beijing, 100850, China

[2]Department of Traditional Chinese Medicine and Neuroimmunopharmacology, Beijing Institute of Pharmacology and Toxicology, Beijing, 100850, China

[3]International Cooperation Laboratory on Signal Transduction, Eastern Hepatobiliary Surgery Institute, Second Military Medical University, Shanghai, 200433, China

[4]Department of Biochemistry and Molecular Biology, Beijing Institute of Radiation Medicine, Beijing, 100850, China

[5]Department of Pharmacy, No.451 hospital of People's Liberation Army, Xi'an, 710065, China

---

[*]Corresponding author. Xiaochen Bo, E-mail:boxc@bmi.ac.cn; Shengqi Wang, E-mail:sqwang@bmi.ac.cn; Hui Bai, E-mail:huibai13@hotmail.com. Tel: 86-10-66931422. Address: Beijing Institute of Radiation Medicine, No.27 Taiping Road, Haidian District, Beijing, 100850, China.

Supplementary Information

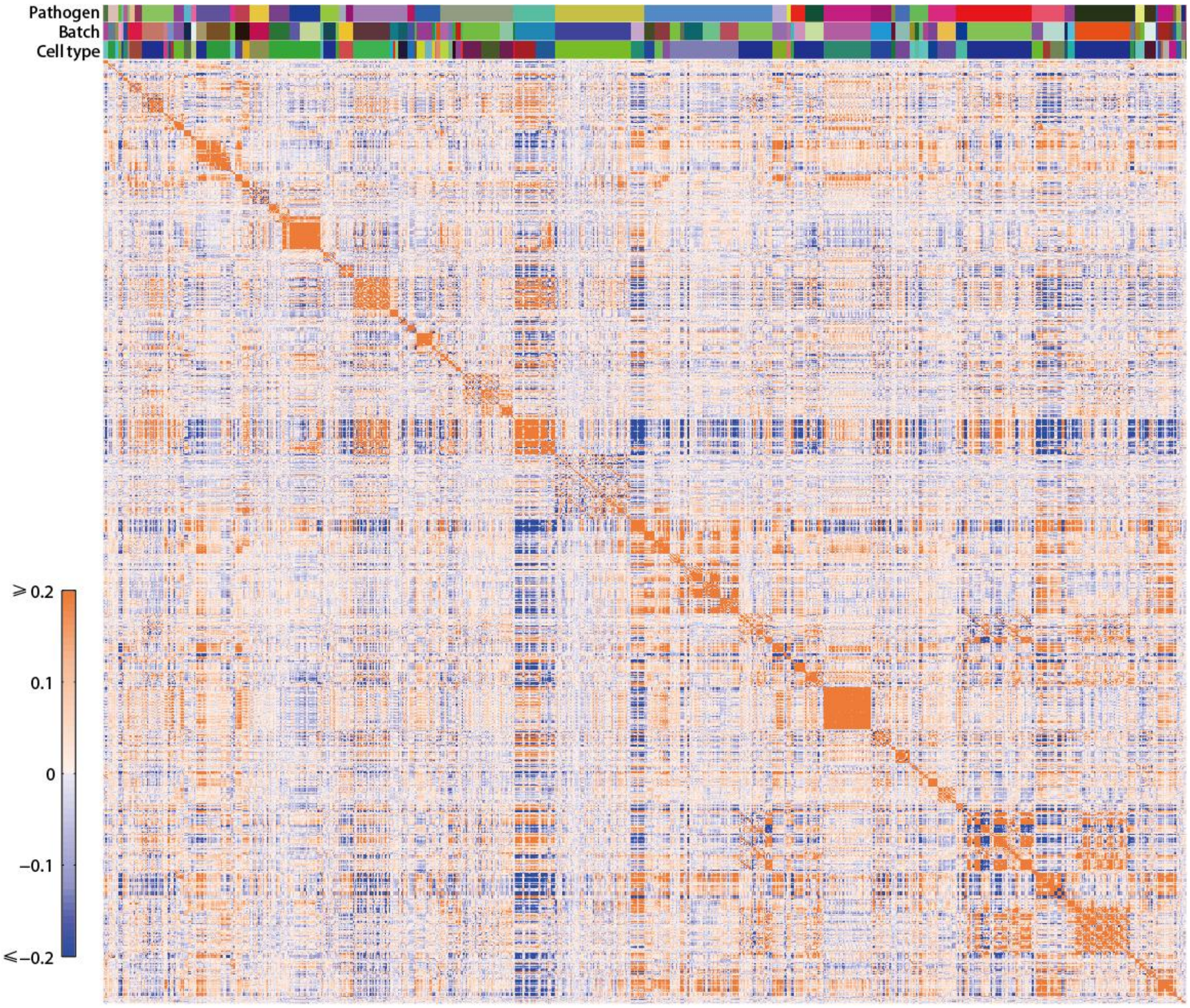

Figure S1. Heat map representation of the spearman correlation coefficients for 893 phonotype ranking lists (PRLs). PRL is a list of ~22160 host cellular genes ranked according to their differential expression upon infection relative to the control. A total of 893 PRLs were collected to represent host transcriptional response (HTR) of 50 different pathogens across different cell types and from different labs. Gene expression profiles of different pathogen types are annotated as differentially colored bars above the heat map. Under each pathogen type, the different cell types and resource providers included in this study are correspondingly clustered and annotated as differentially colored bars above the heat map. Squares in the heat map are differentially colored according to the value of spearman correlation coefficients, with orange representing positive correlation and blue representing negative correlation. As shown in the diagonal, the correlation coefficients among gene expression profiles of a specific pathogen type is significantly higher than those

of different pathogen types (*P* value < 10^{-100}, performed by two sample t test). The original data were provided in Supplementary Data S1.

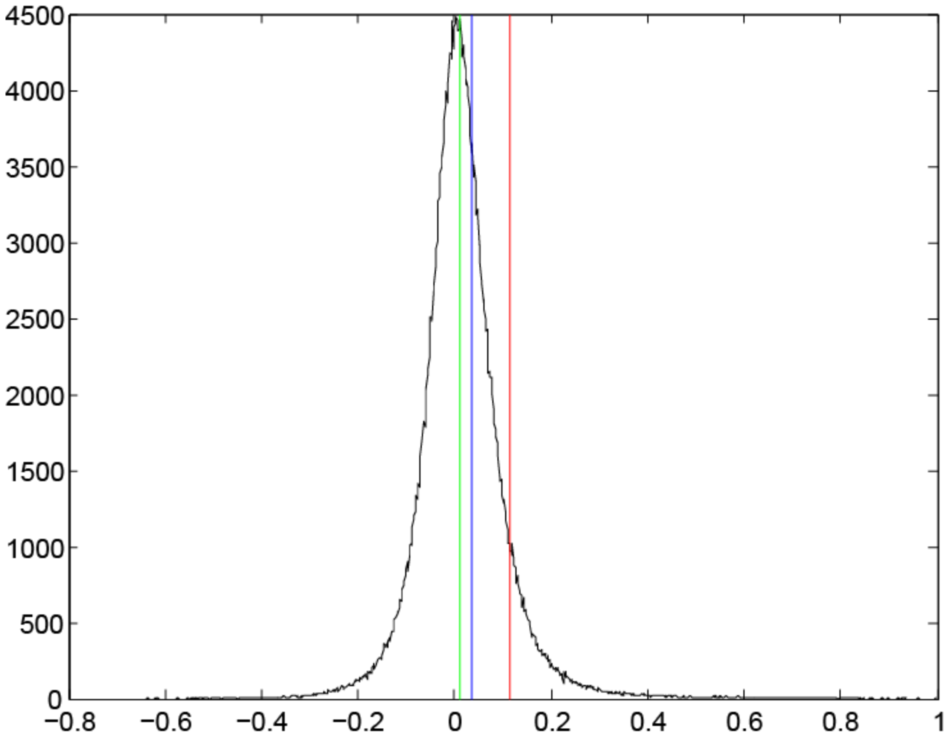

Figure S2. The distribution of spearman correlation coefficients for 893 phonotype ranking lists (PRLs). The mean spearman correlation coefficients of PRLs from same pathogens were 0.1164 as ploted in red line, and that of PRLs from different pathogens were 0.0108 as ploted in green line, and that of PRLs from same cells but different pathogens were 0.0349 as ploted in blue line.

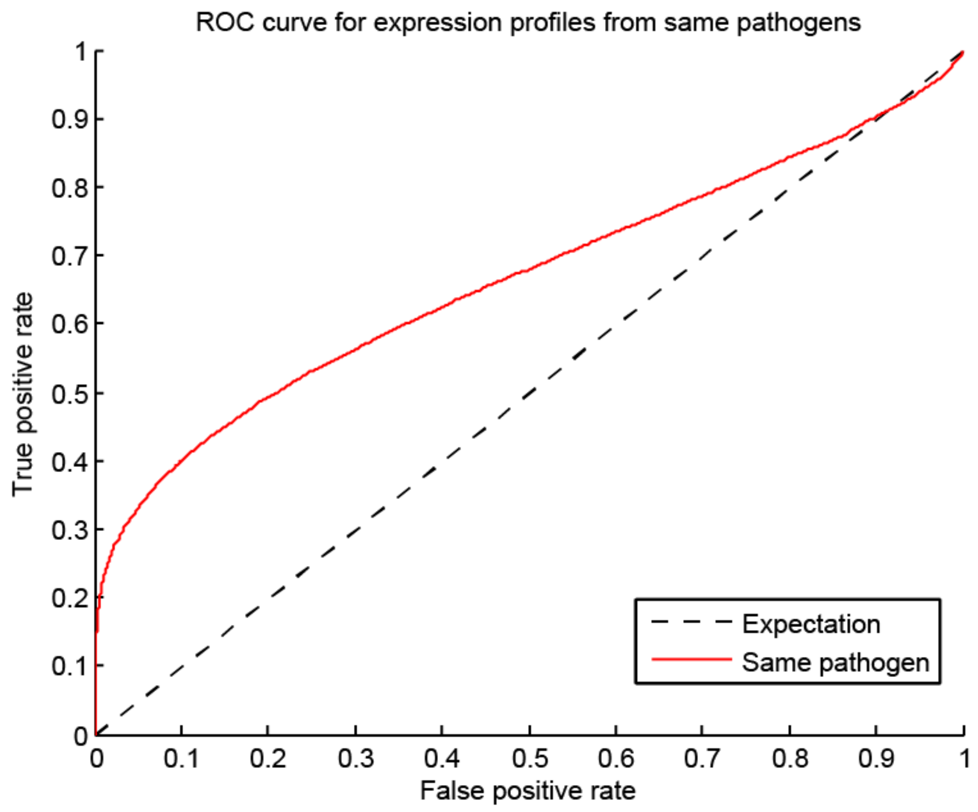

Figure S3. ROC curve for expression profiles from same pathogens. The corresponding area under curve (AUC) is 0.6625.

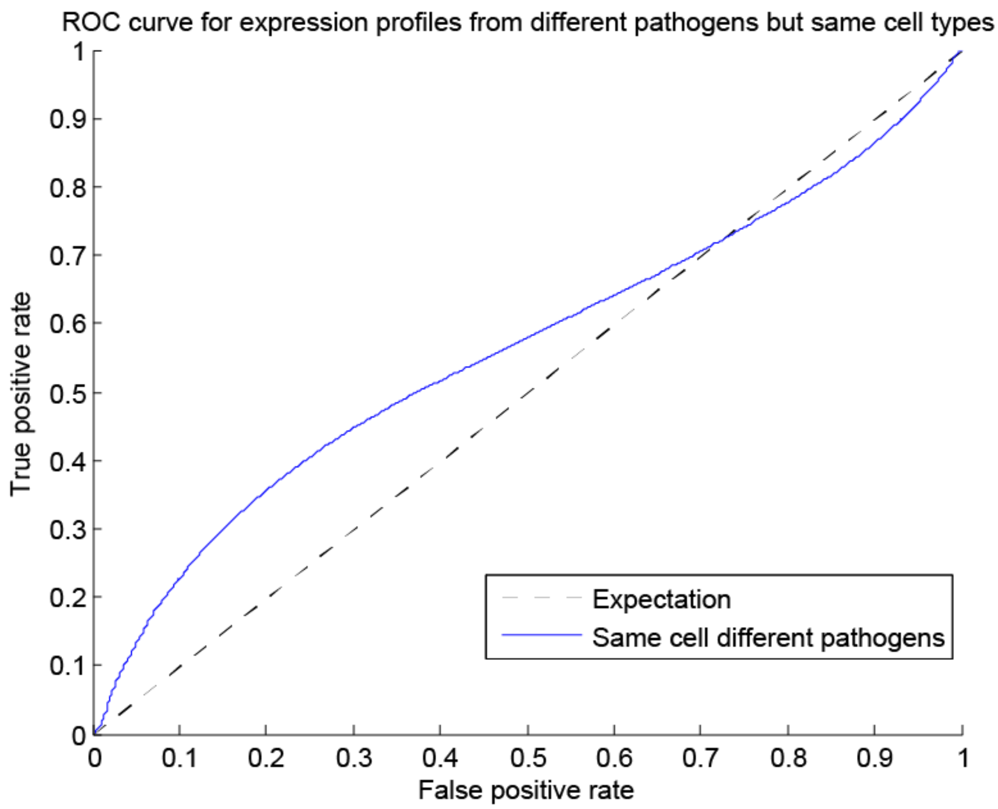

Figure S4. ROC curve for expression profiles from same cells but different pathogen infection. The corresponding area under curve (AUC) is 0.5614.

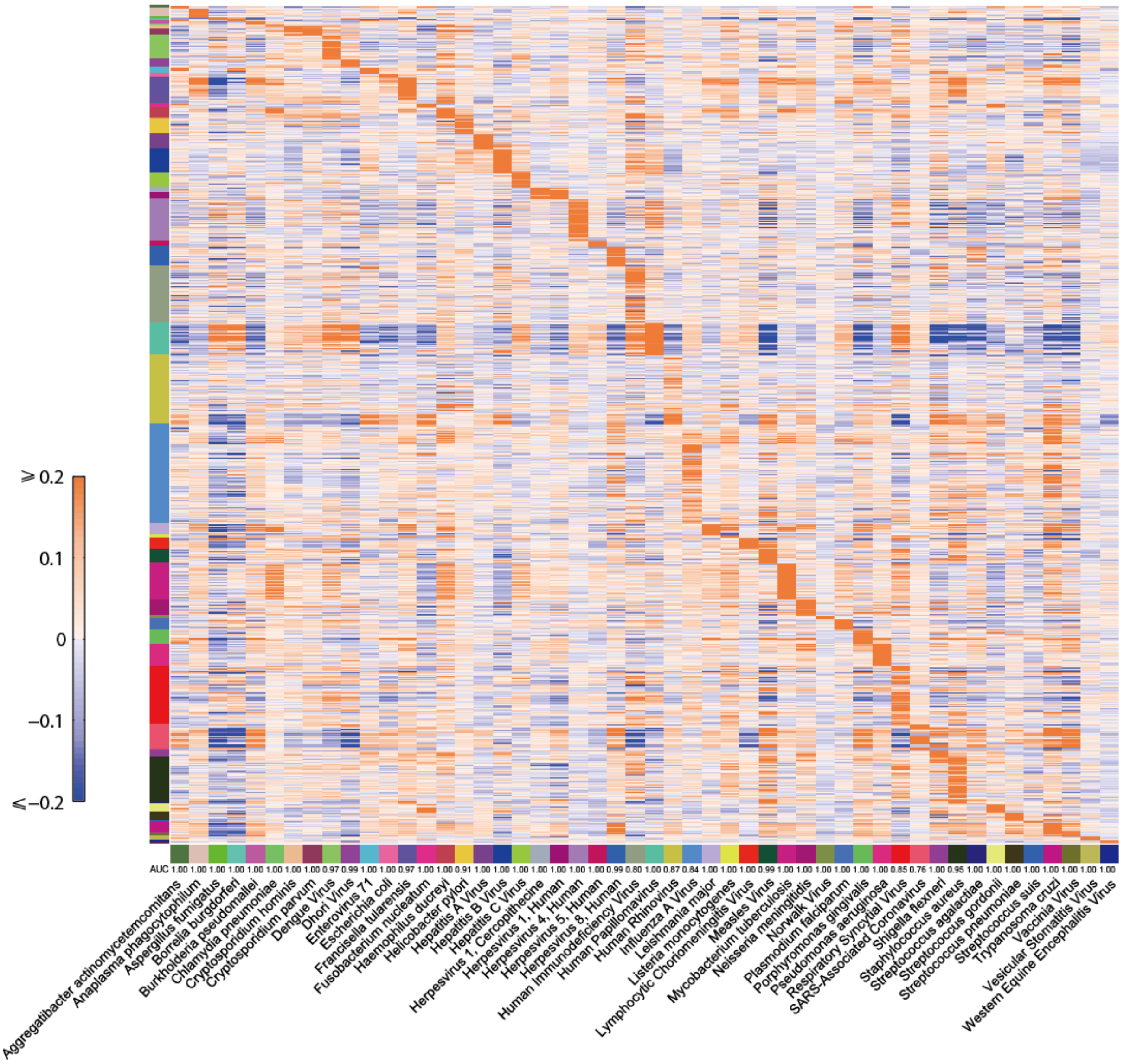

Figure S5. Heat map representation of the spearman correlation coefficients between the 50 merged phenoptype rank lists (mPRLs) and 893 individual PRLs for 50 different pathogens. PRL is a list of ~22160 host cellular genes ranked according to their differential expression upon infection relative to the control. mPRL was computed by merging all the PRLs referring to the same pathogen with a procedure based on a hierarchical majority-voting scheme, where genes consistently up- or down-regulated across each individual PRL are placed at the top or bottom of the merged PRL. 50 mPRLs are horizontal listed and annotated as differentially colored bars under the heat map. A total of 893 PRLs that represent host transcriptional response (HTR) of 50 different pathogens across different cell types and from different labs, are vertically listed and annotated according to belonged pathogen type as differentially colored bars on the left of the heat map. Squares in the heat

map are differentially colored according to the value of spearman correlation coefficient, with orange representing positive correlation and blue representing negative correlation. As shown in the diagonal, for each pathogen type, the mPRL shows high correlation coefficients with individual PRL (*P* value = 0, performed by two sample t test). And for each pathogen type, the area under curve (AUC) obtained with corresponded individual component PRLs as benchmarks was listed below corresponded heatmap column. The merged PRLs and Spearman correlation coefficients between individual PRLs and mPRLs were provided in Supplementary Data S2 and S3.

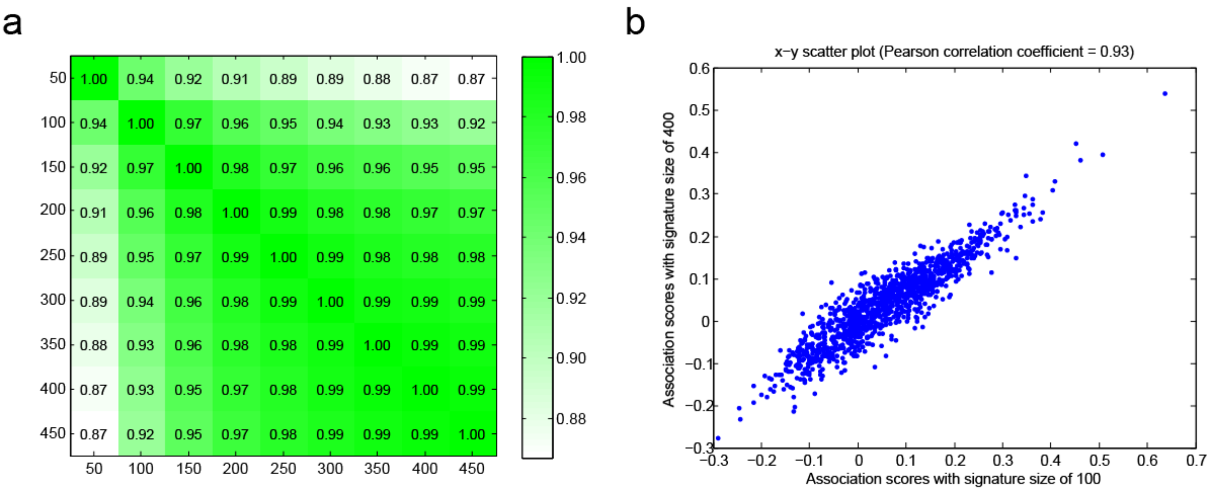

Figure S6. Pearson correlation coefficients of 1225 pairs of Host transcriptional response (HTR) relations calculated with signature size ranging from 50 to 450 genes at the interval of 50 genes. (**a**) The heatmap representation of the correaltion coefficients between 1225 pairwise HTR association scores caculated at different parameters of signature size. (**b**) The x-y scatter plot of 1225 pairwise HTR association scores obtained with signature size at 100 and 400. This plot was used as an example demonstration of the Pearson correlation coefficients calculated in the heatmap. For each point representing one of the 1225 pairwise HTR association scores of 50 pathogens, the x coordinate means the association scorecalculated at the signature size of 100 gene, and the y coordinate means that at the signature size of 400 gene. Together, these results indicated high correlations among 1225 pairwise HTR association scores obtained at different sinature sizes, and therefore the more liberal setting of value for this paramete. The Association Scores between 50 mPRLs generated with different signature sizes were provided in Supplementary Data S4.

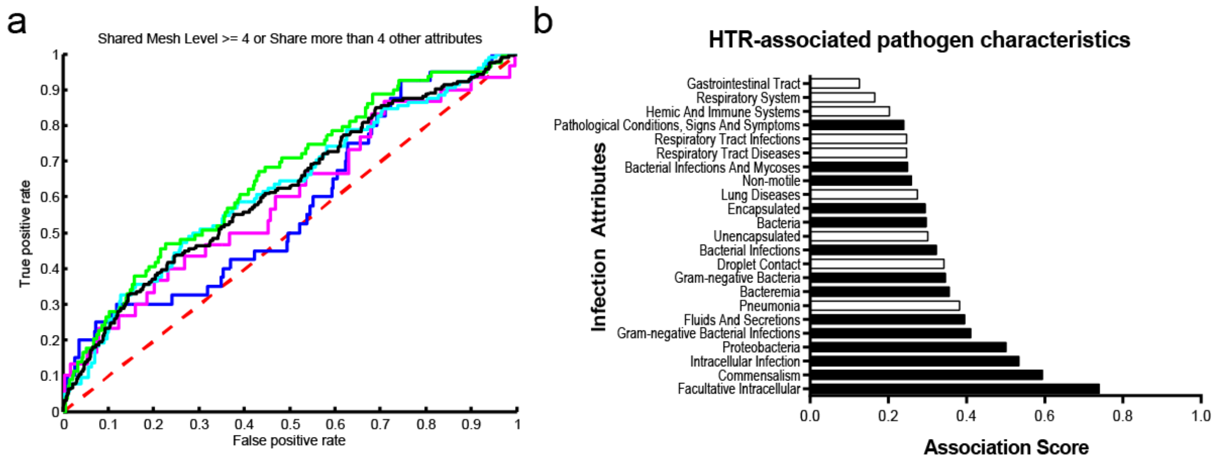

Figure S7. Validation of relations between host transcriptional responses (HTRs) and infection attributes. (**a**) Receiver Operator Characteristic (ROC) curves were generated using pathogens with high infection attribute similarities as benchmarks. The curve color are related to benchmarks as followed, 1) red dash line: expectation at random classification; 2) blue line (AUC = 0.574): benchmarks sharing four or more levels of MeSH Code for organisms; 3) purple line (AUC = 0.592): benchmarks sharing four or more levels MeSH Code for implicated cell, tissue or organ; 4) cyan line (AUC = 0.624): benchmarks sharing four or more levels of manifestation of infectious disease; 5) green line (AUC = 0.656): benchmarks sharing four or more of infections attributes designated as laboratory and clinical characteristics; 6) black line (AUC = 0.620): benchmarks meeting any of the above requirements. The Receiver Operator Characteristic (ROC) curve for HTR simi-larities between 50 pathogens showed limited predictive sensitivity. Especially for benchmarks that represent similar pathogen species (AUC = 0.574) and similar cell tropism (AUC = 0.592), the predictive sensitivity is only above the threshold. And the AUC for each benchmark was lower than 0.7, indicating that HTR similarity was not simply closely related to the benchmarks for each major category of infection attributes above. (**b**) Association scores of HTR-associated infection attributes with *FDR* value less than 0.01, and those overlap with community-specific infection attributes were highlighted in black.

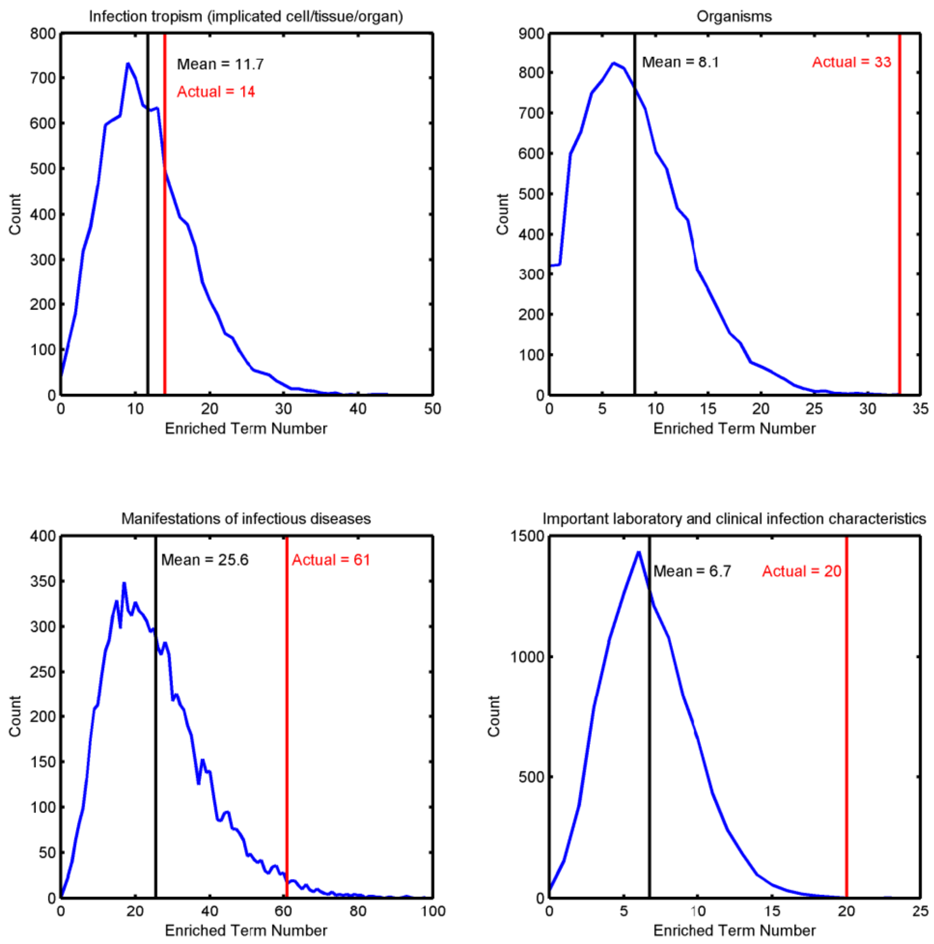

Figure S8. Distribution of the number of community enriched infection attribute terms with respect to that of 10,000 random permutations. The blue line represents the distribution pattern of enriched term numbers at random permutations. The red line represents the actual number of community enriched infection attributes. The percentage of outnumbered random cases were 28.70% for Infection-implicated cell/tissue/organ, 0.00% for Organisms, 1.65% for Manifestations of infectious diseases, and 0.01% for Important laboratory and clinical infection characteristics (Supplementary Tables S4).

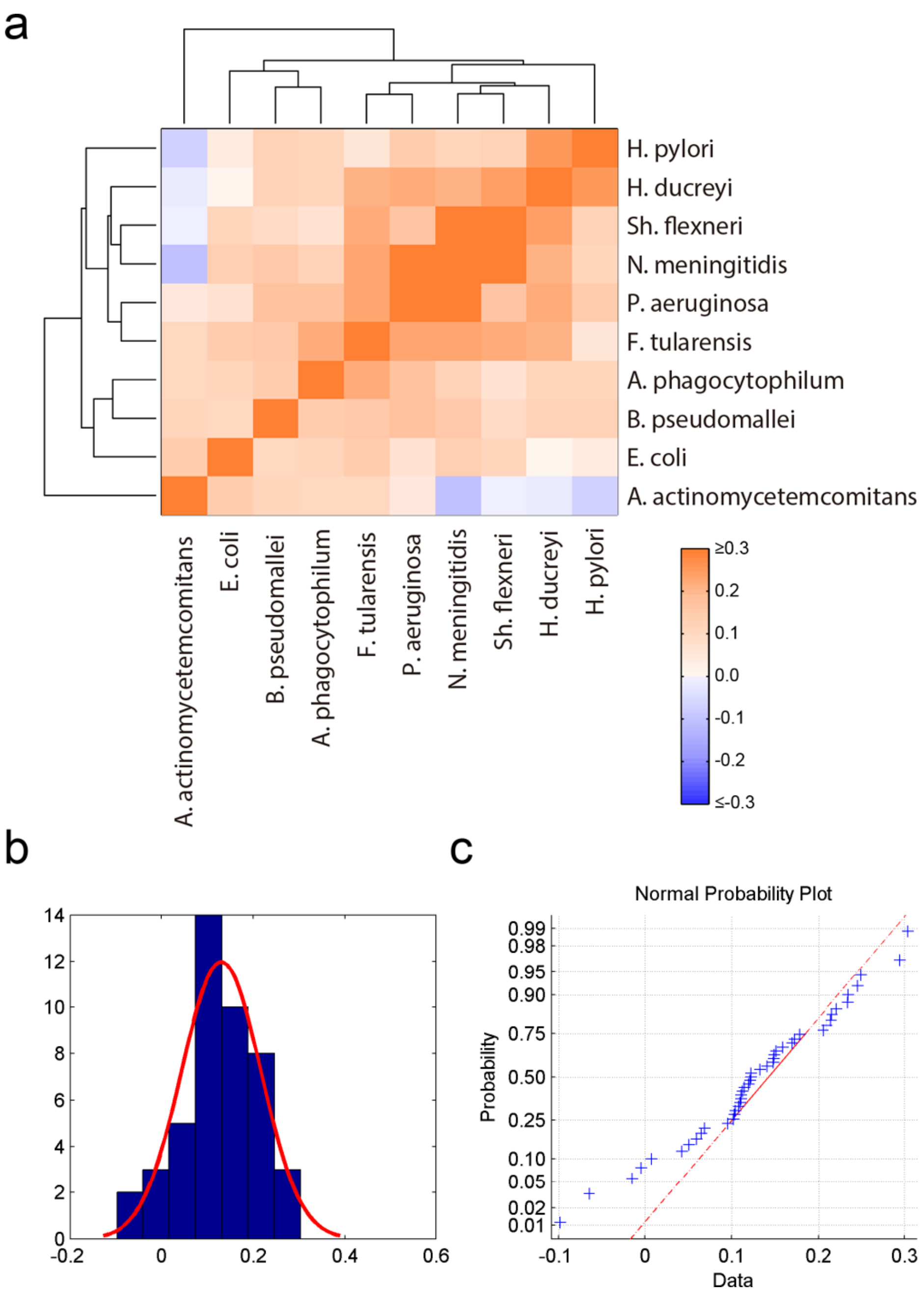

Figure S9. Host transcriptional response (HTR) relations among proteobacteria. (**a**) Heat map presentation of HTR relations among ten proteobacteria in the HTR Community. Each pathogen's HTR was represented as merged phenotype rank list (mPRL) that combines the expression changes across different experimental settings (i.e., pathogen strain/subtype, infected cell line and laboratory). HTR relations was represented as an "association score", and computed with mPRLs of each proteobacteria pair. The order of proteobacteria-to-proteobacteria HTR relations is determined by hierarchical clustering. The color scale indicates the value range of association score (i.e., from +1 to −1), with red

representing "positive association" and blue representing "negative association". (**b-c**) The distribution of association scores of 45 HTR pairs of proteobacteria as approximated to normal distributions. The distribution pattern is in accordance with Gaussian distribution, and presents as mean and standard deviations, i.e., 0.13 ± 0.0861, which deviated their expected center 0 (*P*-value = 1.066×10^{-26}, performed by t-test), indicating significantly similar HTRs among proteobacteria.

Supplementary Table S1. Gene expression profiles datasets of host transcriptional responses to 50 pathogens used in the study.

Pathogen type	Organisms	Mesh term	Strain type or Subspecies[a]	Infection model			GSE NO.	Platform
				in vitro/ in vivo	cell or tissue resource	types		
bacteria	*Aggregatibacter actinomycetemcomitans*	B03.440.450.009.050 B03.660.250.550.050.050	—	in vitro	Homo sapiens	HIGK cells	GSE9723	GPL96
	Anaplasma phagocytophilum	B03.440.040.050.600 B03.660.050.020.050.600	—	in vitro	Homo sapiens	Polymorphonuclear leukocytes	GSE2405	GPL96
			—	in vitro	Homo sapiens	NB4 promyelocytic cells	GSE2600	GPL570
	Borrelia burgdorferi	B03.440.425.410.711.193.150.125 B03.851.595.193.150.125	—	in vitro	Homo sapiens	HUVEC	GSE6092	GPL570
	Burkholderia pseudomallei	B03.440.400.425.251.100.600 B03.660.075.077.100.600	—	in vitro	Homo sapiens	THP-1	GSE7577	GPL96
	Chlamydia pneumoniae	B03.440.190.190.230.249	—	in vitro	Homo sapiens	Dendritic cells derived from human monocytes	GSE12806	GPL570
	Escherichia coli	B03.440.450.425.325.300 B03.660.250.150.180.100	Strain UTI89 Strain MG1655	in vitro	Homo sapiens	Purified human neutrophils	GSE18810	GPL570
	Francisella tularensis	B03.440.400.425.340.590 B03.660.250.200.750	Subspecies novicida Subspecies Schu S4 isolate	in vitro	Homo sapiens	PBMC	GSE12108	GPL570
			Strain LVS	in vitro	Homo sapiens	Polymorphonuclear leukocytes	GSE37416	GPL570
	Fusobacterium nucleatum	B03.370.250.500 B03.440.425.410.420.500	—	in vitro	Homo sapiens	HIGK cells	GSE6927	GPL96
	Haemophilus ducreyi	B03.440.450.600.450.125 B03.660.250.550.290.125	—	in vivo	Homo sapiens	Skin biopsy Dendritic Cells	GSE5547	GPL570
	Helicobacter pylori	B03.440.500.550 B03.660.150.280.550	—	in vivo	Homo sapiens	Gastric mucosa	GSE5081	GPL570
	Listeria monocytogenes	B03.510.100.500.500 B03.510.460.400.410.485.500	—	in vitro	Homo sapiens	Dendritic cells	GSE9946	GPL96
	Mycobacterium tuberculosis	B03.510.024.049.525.500.702 B03.510.460.400.410.552.552.702	Strain R1.4 Strain R17.1 Strain ZA9.2 Strain ZA9.4 Strain R19.4 Strain CHN50.1 Strain MAD2.1 Strain CHN50.2 Strain R17.3	in vitro	Homo sapiens	THP-1	GSE29628	GPL570

			Strain R19.5<br>Strain H37Rv<br>Strain MAD2.2					
	Neisseria meningitidis	B03.440.400.425.550.550.641<br>B03.660.075.525.520.500	Strain MC58_WT<br>Strain MC58_pilD<br>Strain MC58_frpC/frpA	in vitro	Homo sapiens	HUVEC	GSE4646	GPL96
	Porphyromonas gingivalis	B03.140.094.625.515<br>B03.440.425.410.194.625.515	—	in vitro	Homo sapiens	HIGK cells	GSE12121	GPL570
			—	in vitro	Homo sapiens	HTR8	GSE19810	GPL570
			Strain 3D7	in vitro	Homo sapiens	CD34+-derived Hematopoietic stem cells	GSE24897	GPL570
			—	in vitro	Homo sapiens	HIGK cells	GSE9723	GPL96
	Pseudomonas aeruginosa	B03.440.400.425.625.625.100<br>B03.660.250.580.590.050	—	in vitro	Homo sapiens	CFBE41o	GSE30439	GPL570
			Strain FRD1<br>Strain FRD440<br>Strain FRD875<br>Strain FRD1234	in vitro	Homo sapiens	Calu-3 human lung epithelial cells	GSE923	GPL96
	Shigella flexneri	B03.440.450.425.850.450<br>B03.660.250.150.730.210	Strain M90T	in vitro	Homo sapiens	Caco-2 intestinal epithelial cell line	GSE6082	GPL96
			Strain non-invasive<br>Strain invasive<br>Strain MxiE mutant	in vitro	Homo sapiens	Intestinal tissue	GSE8636	GPL96
	Staphylococcus aureus	B03.300.390.400.800.750.100<br>B03.510.100.750.750.100<br>B03.510.400.790.750.100	—	in vivo	Homo sapiens	PBMC	GSE16129	GPL96
			—	in vitro	Homo sapiens	Polymorphonuclear leukocytes	GSE2405	GPL96
	Streptococcus agalactiae	B03.510.400.800.872.100<br>B03.510.550.737.872.100	—	in vitro	Homo sapiens	HCAEC	GSE15495	GPL570
	Streptococcus gordonii	B03.510.400.800.872.260<br>B03.510.550.737.872.260	—	in vitro	Homo sapiens	HIGK cells	GSE12121	GPL570
			—	in vitro	Homo sapiens	HIGK cells	GSE6927	GPL96
	Streptococcus pneumoniae	B03.510.400.800.872.550<br>B03.510.550.737.872.550	Strain D39<br>Strain G54<br>Strain TIGR4	in vitro	Homo sapiens	Human pharyngeal epithelial cells	GSE8527	GPL570
	Streptococcus suis	B03.510.400.800.872.750<br>B03.510.550.737.872.750	Streptcoccus suis 2 (SS2) strain SC19	in vitro	Homo sapiens	Human monocytic cells	GSE20508	GPL570

virus	Dengue Virus	B04.820.250.350.270<br>B04.909.777.310.350.270	—	in vivo	Homo sapiens	PBMC	GSE18090	GPL570
			—	in vitro	Homo sapiens	Primary human umbilical vein endothelial cells	GSE34628	GPL570
			dengue 2 virus, New Guinea C	in vitro	Homo sapiens	HUVEC<br>monocytes<br>B cells	GSE9378	GPL96
	Dhori Virus	B04.820.545.850<br>B04.909.777.545.850	—	in vitro	Homo sapiens	Bronchial epithelial cell line 2B4	GSE17400	GPL570
	Enterovirus 71	B04.820.565.284.180<br>B04.909.777.618.284.180	—	in vitro	Homo sapiens	Human rhabdomyosarcoma cell line	GSE15323	GPL570
	Hepatitis A Virus	B04.450.420.410<br>B04.820.565.400.410<br>B04.909.777.618.400.410	—	in vivo	Pan troglodytes	Liver cells	GSE27850	GPL570
	Hepatitis B Virus	B04.280.375.650.425<br>B04.450.390.650.425<br>B04.909.204.340.650.425	—	in vivo	Homo sapiens	Liver cells	GSE14668	GPL570
			—	in vivo	Homo sapiens	Liver cells	GSE38941	GPL570
	Hepatitis C Virus	B04.450.410<br>B04.820.250.475<br>B04.909.777.310.475	Genotype 1	in vivo	Homo sapiens	Human hepatocytes from human hepatocyte chimeric mouse liver	GSE37715	GPL570
			Genotype 1	in vivo	Homo sapiens	PBMC	GSE40184	GPL96
			—	in vivo	Homo sapiens	Liver cells	GSE7741	GPL570
	Herpesvirus 1, Cercopithecine	B04.280.382.100.750.350<br>B04.909.204.382.100.750.350	Strain E2490	in vitro	Homo sapiens	Human foreskin fibroblast cells	GSE4521	GPL96
	Herpesvirus 1, Human	B04.280.382.100.750.390<br>B04.909.204.382.100.750.390	Strain MacIntyre	in vitro	Homo sapiens	Human foreskin fibroblast cells	GSE4521	GPL96
	Herpesvirus 4, Human	B04.280.382.400.500.450<br>B04.909.204.210.400.500.400<br>B04.909.204.382.400.500.400<br>B04.909.574.204.500.500.400	—	in vivo	Homo sapiens	Nasopharynx	GSE12452	GPL570
			—	in vitro	Homo sapiens	EBV-positve Gamma-delta T cell line SNT-8<br>EBV-positve Gamma-delta T cell line SNT-13<br>EBV-positve Gamma-delta T cell line SNT-15<br>Gamma-delta T cells	GSE13906	GPL570
			Awia-BL	in vitro	Homo sapiens	Burkitt lymphoma cell line	GSE42867	GPL570
			—	in vitro	Homo	A549 cells	GSE5450	GPL96

				sapiens			
Herpesvirus 5, Human	B04.280.382.150.150<br>B04.909.204.382.150.150	Towne strain (ATCC#VR977)	in vitro	Homo sapiens	Human neural stem cell	GSE19345	GPL570
Herpesvirus 8, Human	B04.280.382.400.700.330<br>B04.909.204.210.400.700.330<br>B04.909.204.382.400.700.330<br>B04.909.574.204.500.700.330	—	in vitro	Homo sapiens	Primary human dermal endothelial cells	GSE1377	GPL96
		—	in vitro	Homo sapiens	Lymphatic endothelial cells<br>blood vessel endothelial cells	GSE16354	GPL570
		—	in vitro	Homo sapiens	Lymphatic endothelial cells	GSE22522	GPL570
		—	in vitro	Homo sapiens	EA.hy. 926re	GSE33984	GPL570
		—	in vitro	Homo sapiens	Pulmonary microvascular entdothelial cells	GSE6489	GPL570
Human Immunodeficiency Virus	B04.820.650.589.650.350<br>B04.909.777.731.589.650.350	—	in vivo	Homo sapiens	Subcutaneous abdominal adipose tissue	GSE19811	GPL96
		HIV-1	in vitro	Homo sapiens	Jurkat cells	GSE2504	GPL96
		—	in vivo	Homo sapiens	Jejunal tissue collected by upper endoscopy	GSE28177	GPL570
		—	in vivo	Homo sapiens	CD11c+ Myeloid Dendritic Cells	GSE42058	GPL570
		HIV-1	in vitro	Homo sapiens	CRL-2615	GSE42291	GPL570
		—	in vivo	Homo sapiens	CD4+ T cells<br>CD8+ T cells	GSE6740	GPL96
		HIV-1	in vivo	Homo sapiens	CD4+ T cells	GSE9927	GPL570
Human Papillomavirus	B04.280.535[b]<br>B04.909.204.210.655 [b]<br>B04.909.574.204.655 [b]<br>B04.909.624 [b]	—	in vitro	Homo sapiens	SCC4<br>SCC74A<br>SCC47<br>CaSki	GSE24089	GPL570
		HPV-16<br>HPV-18<br>HPV-31<br>HPV-33<br>HPV-35<br>HPV-58<br>HPV-66	in vivo	Homo sapiens	Cervical cancer and normal cells<br>Head and neck cancer and normal cells	GSE6791	GPL570
Human Rhinovirus	B04.820.565.775<br>B04.909.777.618.775	—	in vivo	Homo sapiens	Nasal epithelium	GSE11348	GPL570

			—	in vitro	Homo sapiens	Human primary bronchial epithelial cells	GSE13396	GPL570
	Influenza A Virus	B04.820.545.405.400<br>B04.909.777.545.405.400	Strain PR8<br>Strain FPV<br>Strain H5N1	in vitro	Homo sapiens	HUVEC	GSE13637	GPL570
			Strain PR8	in vitro	Homo sapiens	Primary alveolar macrophages alveolar epithelial cells	GSE30723	GPL570
			Strain A/WSN/33(H1N1)	in vitro	Homo sapiens	A549 cells	GSE31469	GPL570
			Strain A/WSN/33(H1N1)	in vitro	Homo sapiens	A549 cells	GSE31470	GPL570
			Strain A/Duck/Malaysia/01(H9N2)	in vitro	Homo sapiens	A549 cells	GSE31471	GPL570
			Strain A/duck/Malaysia/F118/08/2004(H5N2)	in vitro	Homo sapiens	A549 cells	GSE31472	GPL570
			Strain A/Singapore/478/2009 (pH1N1)	in vitro	Homo sapiens	A549 cells	GSE31518	GPL570
			—	in vivo	Homo sapiens	PBMC	GSE34205	GPL570
	Lymphocytic Choriomeningitis Virus	B04.820.057.070.100.550<br>B04.909.777.080.070.100.550	Strain LCMV-WE<br>Strain LCMV-ARM	in vivo	Macaca mulatta	Liver cells	GSE12254	GPL570
			Strain LCMV-WE<br>Strain LCMV-ARM	in vivo	Macaca mulatta	PBMC	GSE5790	GPL570
	Measles Virus	B04.820.455.600.650.500.500<br>B04.909.777.455.600.650.500.500	—	in vivo	Homo sapiens	PBMC	GSE5808	GPL96
	Norwalk Virus	B04.820.095.550.500<br>B04.909.777.162.550.500	—	in vitro	Homo sapiens	Huh-7	GSE15520	GPL570
	Respiratory Syncytial Virus	B04.820.455.600.670.600.750<br>B04.909.777.455.600.670.600.750	—	in vitro	Homo sapiens	Isolated conventional and plasmacytoid dendritic cells	GSE24132	GPL570
			—	in vivo	Homo sapiens	PBMC	GSE34205	GPL570
	Severe Acute Respiratory Syndrome-Associated Coronavirus	B04.820.504.540.150.750<br>B04.909.777.500.540.150.750	—	in vitro	Homo sapiens	Bronchial epithelial cell line 2B4	GSE17400	GPL570
			Strain rSARS-CoV<br>Strain rSARS-CoV-ΔE	in vivo	Chlorocebus aethiops	Vero E6 cells<br>MA-104 cells	GSE30589	GPL570
	Vaccinia Virus	B04.280.650.160.650.900<br>B04.909.204.783.160.650.900	Strain wild type<br>Strain E3L mutant	in vitro	Homo sapiens	HeLa cell line	GSE11238	GPL570
	Vesicular Stomatitis Virus	B04.820.455.750.900[c]<br>B04.909.777.455.750.900[c]	—	in vitro	Homo sapiens	Human Astrocytes	GSE2449	GPL96
	Western Equine	B04.820.850.054.360	—	in vitro	Homo	BE(2)-C cell line	GSE16451	GPL570

	Encephalitis Virus	B04.909.777.270.440<br>B04.909.777.923.054.360			sapiens			
fungi	*Aspergillus fumigatus*	B01.300.381.081.295	—	in vitro	Homo sapiens	Human dendritic cells	GSE6965	GPL570
parasite	*Cryptosporidium hominis*	B01.043.075.189.250.150.160[d]	—	in vitro	Homo sapiens	Ileal tissue	GSE7268	GPL570
	Cryptosporidium parvum	B01.043.075.189.250.150.160.170	—	in vitro	Homo sapiens	Ileal tissue	GSE7268	GPL570
	Leishmania major	B01.268.475.868.488.405	—	in vitro	Homo sapiens	Human dendritic cells	GSE42088	GPL570
	Plasmodium falciparum	B01.043.075.380.611.561	—	in vitro	Homo sapiens	growth factor-mobilized CD34+ hematopoietic stem cells	GSE24849	GPL570
	Trypanosoma cruzi	B01.268.475.868.887.140	—	in vitro	Homo sapiens	Human microvascular endothelial cells/<br>Human vascular smooth muscle cells	GSE13791	GPL570
			—	in vitro	Homo sapiens	HeLa cell line	GSE7047	GPL570

Abbreviations: HIGK cells, human immortalized gingival keratinocyte human oral epithelial cells; THP1, a human monocytic cell line derived from an acute monocytic leukemia patient. HUVEC, human umbilical vein endothelial cells; HCAEC, human coronary artery endothelial cells; PBMC, peripheral blood mononuclear cells; EA.hy. 926, a hybrid cell line established by fusing a human umbilical vein endothelial cell with a human carcinoma cell line. Vero E6 cells, isolated from kidney epithelial cells extracted from an African green monkey (Cercopithecus aethiops); MA-104 cells, non-human, epithelial monkey kidney Cells

[a] "—", no specific declaration on strain information in the dataset descriptions.

[b]The MeSH tree number refers to *Papillomaviridae* for multiple species of *Papillomaviridae* included.

[c]The MeSH tree number refers to the pathogen's genus due to inadequate information about its species in dataset descriptions.

[d]The MeSH tree number refers to the pathogen's genus for there was no MeSH tree number designated for its specie.

Supplementary Table S2. Infection attributes collected for 50 pathogens in the host transcriptional response (HTR) Community.

Pathogens	Infection implicated cells/tissues/organs	Infection implicated cells/tissues/organs Mesh code	Manifestations of infectious diseases	Manifestations of infectious diseases Mesh code	Important laboratory and clinical infection characteristics
Aggregatibacter actinomycetemcomitans	oral cavity	A01.456.505.631; A03.556.500; A14.549	periodontitis	C07.465.714.533	commensalism; extracellular; immunosuppression; non-motile; direct physical contact; saliva; vector-borne transmission; oral flora
Anaplasma phagocytophilum	neutrophils	A11.118.637.415.583; A11.627.340.583; A11.733.689; A15.145.229.637.415.583; A15.382.490.315.583; A15.382.680.689	ehrlichiosis	C01.252.400.054.160; C01.252.400.825.200	obligate intracellular; intracellular infection; encapsulated
Aspergillus fumigatus	lung; bronchus; endosome	A04.411; A04.411.125; A11.284.430.214.190.875.190.880.337	invasive pulmonary aspergillosis; allergic bronchopulmonary aspergillosis; hypersensitivity	C08.381.472.850.750; C01.539.800.200.383.249.074; C01.703.295.328.249.074; C01.703.513.249.074; C01.703.534.090; C08.381.472.850.500; C08.674.060; C08.730.435.090; C17.800.838.208.416.249.074; C20.543.480.680.085; C20.543	extracellular; opportunity infection immunocompromised; airborne transmission
Borrelia burgdorferi	skin; heart; joint; eye; brain	A17.815; A07.541; A02.835.583; A01.456.505.420; A09.371; A08.186.211	lyme disease; macular degeneration; erythema chronicum migrans; myocarditis; cardiomyopathy; arrythmia; arthritis; arthralgia; meningitis; facial paralysis	C01.252.400.155.569; C01.252.400.825.480; C01.252.847.193.569; C11.768.585.439; C01.252.400.155.569.200; C01.252.400.825.480.400; C01.252.825.310; C01.252.847.193.569.200; C01.539.800.720.310; C17.800.229.200;	extracellular; microaerophilic; animal bite; vector-borne transmission; whole-body distributed

				C17.800.838.765.310; C14.280.238.625; C14.280.238; C14.280.067; C23.550.073; C05.550.114; C05.550.091; C23.888.646.130; C10.228.228.507; C10.228.566; C07.465.327; C10.597.622.214; C23.888.592.636.214	
Burkholderia pseudomallei	lung; intestines; skin; brain; joint; bone marrow	A04.411; A03.556.124; A17.815; A08.186.211; A02.835.583; A15.382.216	melioidosis; abscess; pneumonia; bacteremia	C01.252.400.170.531; C01.539.830.025; C23.550.470.756.100; C08.381.677; C08.730.610; C01.252.100; C01.539.757.100; C23.550.470.790.500.100	chronic infection; encapsulated; motile
Chlamydia pneumoniae	lung; bronchus; pharynx	A04.411; A04.411.125; A03.556.750; A04.623; A14.724	pharyngitis; bronchopneumonia; primary atypical pneumonia	C07.550.781; C08.730.561; C09.775.649; C08.127.509; C08.381.677.127; C08.730.610.127; C01.252.400.610.610.760; C01.252.620.500; C08.381.677.540.500; C08.730.610.540.500	obligate intracellular; intracellular infection; cancer bacteria; oncogenic infection; unencapsulated; motile; respiratory droplets; droplet contact
Cryptosporidium hominis	gastrointestinal tract; lung; liver; gallbladder	A03.556; A04.411; A03.620; A03.159.439	cryptosporidiosis	C03.432.269; C03.701.688.235; C03.752.250.269; C03.752.625.235; C06.405.469.452.269; C22.674.710.235	obligate intracellular; intracellular infection; opportunity infection immunocompromised; fecal-oral
Cryptosporidium parvum	gastrointestinal tract	A03.556	cryptosporidiosis; hepatitis; cholecystitis	C03.432.269; C03.701.688.235; C03.752.250.269; C03.752.625.235; C06.405.469.452.269; C22.674.710.235; C06.552.380; C06.130.564.263	obligate intracellular; intracellular infection; opportunity infection immunocompromised; indirect physical contact
Dengue Virus	blood; white blood cells;	A12.207.152; A15.145;	viremia; dengue fever;	C02.937;	immunosuppression; chronic

	dendritic cells; skin; gastrointestinal tract; throat; muscle; brain; liver	A11.118.637; A15.145.229.637; A15.382.490; A11.066.270; A11.436.270; A15.382.066.270; A15.382.812.260; A17.815; A03.556; A03.556.750; A04.623; A14.724; A02.633; A10.690; A08.186.211; A03.620	dengue hemorrhagic fever; dengue shock syndrome	C23.550.470.790.500.900; C02.081.270; C02.782.350.250.214; C02.782.417.214; C02.081.270.200; C02.782.350.250.214.200; C02.782.417.214.200; C02.081.270.200; C02.782.350.250.214.200; C02.782.417.214.200	infection; enveloped; cytoplasm; vector-borne transmission
Dhori Virus	liver; lung	A03.620; A04.411			enveloped; nucleus; vector-borne transmission
Enterovirus 71	gastrointestinal tract; brain; throat; muscle; nervous system	A03.556; A08.186.211; A03.556.750; A04.623; A14.724; A02.633; A10.690; A08	hand, foot and mouth disease; encephalitis; aseptic meningitis; poliomyelitis; herpangina; conjunctivitis, acute hemorrhagic; myoclonic jerk; headache; fever; vomiting	C02.782.687.359.213.331; C02.182.500; C10.228.140.430; C10.228.228.210.150; C10.228.228.245; C02.182.550.600; C02.587.600; C10.228.228.210.500.600; C10.228.228.507.220; C10.228.228.507.700.600; C02.182.600.700; C02.182.700; C02.782.687.359.764; C10.228.228.210.575.750; C10.228.228.210.650; C10.228.228.618.850; C10.228.854.525.850; C10.228.854.641; C10.668.864; C02.782.687.359.213.466; C02.325.250.250; C02.782.687.359.201; C02.782.687.359.213.165; C11.187.183.240.216; C11.294.800.250.250; C10.597.350.500;	acute infection; unenveloped; cytoplasm; fecal-oral

				C23.888.592.350.500; C10.597.617.470; C23.888.592.612.441; C23.888.646.487; C23.888.119.344; C23.888.821.937	
Escherichia coli	large intestine	A03.556.124.526; A03.556.249.249	suppuration; gastroenteritis; urinary tract infection; bacterial meningitis; hemolytic uremic syndrome; peritonitis; mastitis; septicemia; pneumonia, bacterial; gram-negative bacterial infections	C01.539.830; C23.550.470.756; C06.405.205; C01.539.895; C12.777.892; C13.351.968.892; C01.252.200.500; C10.228.228.180.500; C10.228.228.507.280; C12.777.419.936.463; C13.351.968.419.936.463; C15.378.071.141.610; C15.378.140.855.925.500; C01.539.463.600; C06.844.640; C13.703.844.603; C17.800.090.968; C01.539.757; C23.550.470.790.500; C01.252.620; C08.381.677.540; C08.730.610.540; C01.252.400	commensalism; extracellular; facultative anaerobic; encapsulated; unencapsulated; motile; fecal-oral; gut flora; intestinal flora
Francisella tularensis	lung; liver; spleen; lymph nodes; bone marrow; macrophage	A04.411; A03.620; A10.549.700; A15.382.520.604.700; A10.549.400; A15.382.520.604.412; A15.382.216; A11.329.372; A11.627.482; A11.733.397; A15.382.680.397; A15.382.812.522	tularemia; primary atypical pneumonia	C01.252.400.825.900; C01.252.400.939; C01.252.400.610.610.760; C01.252.620.500; C08.381.677.540.500; C08.730.610.540.500	facultative intracellular; intracellular infection; encapsulated; non-motile; vector-borne transmission
Fusobacterium nucleatum	oral cavity; pleural cavity	A01.456.505.631; A03.556.500; A14.549; A01.923.761.800.650	periodontitis; dental plaque	C07.465.714.533; C07.793.208.377	commensalism; encapsulated; non-motile; saliva; vertical transmission; oral flora

Haemophilus ducreyi	genitalia	A05.360	chancroid	C01.252.400.700.433.257; C01.252.810.201; C01.539.778.281.201; C12.294.668.281.201; C13.351.500.711.281.201	facultative intracellular; intracellular infection; facultative anaerobic; encapsulated; unencapsulated; non-motile; sexual contact
Helicobacter pylori	upper gastrointestinal tract; mucus; gastric mucosa; epithelial cells	A03.556.875; A12.200.503; A03.556.875.875.440; A10.615.550.291; A11.436	gastritis; peptic ulcers; stomach neoplasms	C06.405.205.697; C06.405.748.398; C06.405.469.275.800; C06.405.608; C06.405.748.586; C04.588.274.476.767; C06.301.371.767; C06.405.249.767; C06.405.748.789	extracellular; microaerophilic; chronic infection; cancer bacteria; oncogenic infection; highly motile; direct physical contact; stomach flora
Hepatitis A Virus	liver; hepatocytes; kupffer cells	A03.620; A11.436.348; A11.329.372.588; A11.627.482.588; A11.733.397.588; A15.382.680.397.588; A15.382.812.522.588	hepatitis a	C02.440.420; C02.782.687.359.500; C06.552.380.705.422	acute infection; unenveloped; cytoplasm; fecal-oral
Hepatitis B Virus	liver; hepatocytes	A03.620; A11.436.348	hepatitis b; cirrhosis, hepatic; hepatocellular carcinoma	C02.256.430.400; C02.440.435; C06.552.380.705.437; C06.552.630; C04.557.470.200.025.255; C04.588.274.623.160; C06.301.623.160; C06.552.697.160	acute infection; chronic infection; oncovirus; oncogenic infection; enveloped; nucleus; bodily fluids
Hepatitis C Virus	liver; hepatocytes	A03.620; A11.436.348	hepatitis c; cirrhosis, hepatic; hepatocellular carcinoma; esophageal varices; gastric varices; cryoglobulinemia	C02.440.440; C02.782.350.350; C06.552.380.705.440; C06.552.630; C04.557.470.200.025.255; C04.588.274.623.160; C06.301.623.160; C06.552.697.160; C06.405.117.240;	immune system diseases; chronic infection; oncovirus; oncogenic infection; enveloped; cytoplasm; blood; sexual contact

				C06.552.494.414; C06.405.117.240; C06.552.494.414; C14.907.454.140; C15.378.147.780.243; C15.378.463.515.140; C20.683.780.250	
Herpesvirus 1, Cercopithecine	neuron; skin	A08.663; A11.671; A17.815	cns disease	C10.228	acute infection; latent infection; enveloped; nucleus; animal bite; vector-borne transmission
Herpesvirus 1, Human	neuron; skin; mucous membrane; lip	A08.663; A11.671; A17.815; A10.615.550; A01.456.505.631.515; A14.549.336	cold sores	C02.256.466.382.316; C02.825.320.320; C07.465.409.466; C17.800.838.790.320.320	latent infection; enveloped; nucleus; direct physical contact; saliva; oral transmission
Herpesvirus 4, Human	b cells; epithelial cells	A11.063.438; A11.118.637.555.567.562; A15.145.229.637.555.567.562; A15.382.032.438; A15.382.490.555.567.562; A11.436	infectious mononucleosis; glandular fever	C02.256.466.313.400; C15.378.553.381; C15.604.515.516; C20.683.515.515; C02.256.466.313.400; C15.378.553.381; C15.604.515.516; C20.683.515.515	chronic infection; latent infection; oncovirus; oncogenic infection; enveloped; nucleus; saliva; direct physical contact; blood
Herpesvirus 5, Human	salivary glands	A03.556.500.760; A10.336.779; A14.549.760	infectious mononucleosis; glandular fever; mucoepidermoid carcinoma; atherosclerosis	C02.256.466.313.400; C15.378.553.381; C15.604.515.516; C20.683.515.515; C02.256.466.313.400; C15.378.553.381; C15.604.515.516; C20.683.515.515; C04.557.470.200.025.340; C04.557.470.590.340; C14.907.137.126.307	chronic infection; latent infection; opportunity infection immunocompromised; enveloped; nucleus; bodily fluids; vertical transmission; oral transmission
Herpesvirus 8, Human	lymphocytes	A11.118.637.555.567;	kaposi's sarcoma; primary	C02.256.466.860;	chronic infection; acute infection;

		A15.145.229.637.555.567; A15.382.490.555.567	effusion lymphoma; giant lymph node hyperplasia	C04.557.450.795.850; C04.557.645.750; C04.557.386.480.150.592; C15.604.515.569.480.150.592; C20.683.515.761.480.150.592; C15.604.515.245; C20.683.515.250	latent infection; oncovirus; oncogenic infection; opportunity infection immunocompromised; enveloped; nucleus; bodily fluids
Human Immunodeficiency Virus	immune system	A15.382	aids	C02.782.815.616.400.040; C02.800.801.400.040; C02.839.040; C20.673.480.040	chronic infection; enveloped; nucleus; sexual contact; blood; breast milk; vertical transmission
Human Papillomavirus	skin; genitalia; larynx; respiratory tract; keratinocytes	A17.815; A05.360; A04.329; A04; A11.436.397	warts; verruca; warts; cervix cancer; vulva cancer; vagina cancer; penile neoplasms; oropharyngeal neoplasms; anus cancer; oropharyngeal neoplasms	C02.256.650.810; C02.825.810; C02.928.914; C04.925.744; C17.800.838.790.810; C02.256.650.810; C02.825.810; C02.928.914; C04.925.744; C17.800.838.790.810; C02.256.650.810; C02.825.810; C02.928.914; C04.925.744; C17.800.838.790.810; C04.588.945.418.948.850; C13.351.500.852.593.131; C13.351.500.852.762.850; C13.351.937.418.875.850; C04.588.945.418.968; C13.351.500.944.819; C13.351.937.418.968; C04.588.945.418.955; C13.351.500.894.834; C13.351.937.418.937; C04.588.945.440.715; C12.294.260.500; C12.294.494.591; C12.758.409.500; C04.588.443.665.710.684;	chronic infection; acute infection; oncovirus; oncogenic infection; unenveloped; nucleus; direct physical contact; sexual contact; vertical transmission

				C07.550.745.671; C09.647.710.685; C09.775.549.685; C04.588.274.476.411.307.790.040; C06.301.371.411.307.790.040; C06.405.249.411.307.790.040; C06.405.469.491.307.790.040; C06.405.469.860.101.163; C06.405.469.860.180.500.040; C04.588.443.665.710.684; C07.550.745.671; C09.647.710.685; C09.775.549.685	
Human Rhinovirus	nose; respiratory mucosa	A01.456.505.733; A04.531; A09.531; A04.760; A10.615.550.760	common cold	C02.782.687.207; C08.730.162	acute infection; unenveloped; cytoplasm; droplet contact; direct physical contact; indirect physical contact
Influenza A Virus	respiratory tract	A04	influenza, human; fever; cough; pharyngitis; sore throat; myalgia; myalgia; conjunctivitis; dyspnea; pneumonia	C02.782.620.365; C08.730.310; C23.888.119.344; C08.618.248; C23.888.852.293; C07.550.781; C08.730.561; C09.775.649; C07.550.781; C08.730.561; C09.775.649; C05.651.542; C10.597.617.231.249; C10.668.491.525; C05.651.542; C10.597.617.231.249; C10.668.491.525; C11.187.183; C08.618.326; C23.888.852.371; C08.381.677; C08.730.610	acute infection; enveloped; nucleus; droplet contact; viral droplet nuclei transmission
Leishmania major	skin; macrophage; dendritic cells	A17.815; A11.329.372; A11.627.482; A11.733.397; A15.382.680.397; A15.382.812.522; A11.066.270; A11.436.270; A15.382.066.270;	cutaneous leishmaniasis	C03.752.300.500.400; C03.858.560.400; C17.800.838.775.560.400	

		A15.382.812.260			
Listeria monocytogenes	gastrointestinal tract; intestinal epithelium	A03.556; A03.556.124.369; A10.615.550.444	listeriosis; septicemia; meningitis; meningoencephalitis; encephalitis; corneal ulcer; pneumonia; bacterial infection; uterine diseases; bacterial infection; cervix diseases	C01.252.410.514; C01.539.757; C23.550.470.790.500; C10.228.228.507; C10.228.566; C10.228.140.430.550; C10.228.228.245.550; C10.228.228.507.850; C10.228.228.553; C10.228.566.500; C02.182.500; C10.228.140.430; C10.228.228.210.150; C10.228.228.245; C01.539.375.177; C11.204.564.225; C11.294.177; C08.381.677; C08.730.610; C01.252; C13.351.500.852; C01.252; C13.351.500.852.593	facultative intracellular; intracellular infection; unencapsulated; motile; direct physical contact; vertical transmission; fecal-oral
Lymphocytic Choriomeningitis Virus	brain; spinal cord; cerebrospinal fluid	A08.186.211; A08.186.854; A12.207.270.210	aseptic meningitis; encephalitis; meningoencephalitis	C02.182.550.600; C02.587.600; C10.228.228.210.500.600; C10.228.228.507.220; C10.228.228.507.700.600; C02.182.500; C10.228.140.430; C10.228.228.210.150; C10.228.228.245; C10.228.140.430.550; C10.228.228.245.550; C10.228.228.507.850; C10.228.228.553; C10.228.566.500	chronic infection; enveloped; cytoplasm; vector-borne transmission; indirect physical contact
Measles Virus	respiratory tract; oropharynx; eye	A04; A04.623.603; A14.724.603; A01.456.505.420; A09.371	measles	C02.782.580.600.500.500	chronic infection; acute infection; enveloped; cytoplasm; droplet contact; viral droplet nuclei transmission
Mycobacterium	lung; pulmonary alveoli;	A04.411; A04.411.715;	tuberculosis; primary	C01.252.410.040.552.846;	facultative intracellular;

tuberculosis	alveolar macrophage	A11.329.372.600; A11.627.482.600; A11.733.397.600; A15.382.680.397.600; A15.382.812.522.600	atypical pneumonia	C01.252.400.610.610.760; C01.252.620.500; C08.381.677.540.500; C08.730.610.540.500	intracellular infection; cancer bacteria; oncogenic infection; unencapsulated; non-motile; droplet contact; skin flora
Neisseria meningitidis	cerebrospinal fluid; nasopharynx; blood	A12.207.270.210; A04.623.557; A14.724.557; A12.207.152; A15.145	meningitis; septicemia	C10.228.228.507; C10.228.566; C01.539.757; C23.550.470.790.500	facultative intracellular; intracellular infection; encapsulated; unencapsulated; non-motile; droplet contact; conjunctival flora; skin flora; respiratory flora
Norwalk Virus	gastrointestinal tract	A03.556	infectious diarrheal disease; dysentery	C06.405.205.331; C06.405.469.300; C06.405.205.331; C06.405.469.300	chronic infection; acute infection; unenveloped; cytoplasm; fecal-oral; indirect physical contact
Plasmodium falciparum	liver; blood; hepatocytes; erythrocytes	A03.620; A12.207.152; A15.145; A11.436.348; A11.118.290; A11.443.240; A15.145.229.334	malaria, falciparum; cerebral malaria	C03.752.530.650; C03.105.300.500; C03.752.530.620; C03.752.530.650.675; C10.228.228.205.300.500	facultative intracellular; intracellular infection; autoimmune hemolysis; vector-borne transmission
Porphyromonas gingivalis	oral cavity; fibroblast; upper gastrointestinal tract; respiratory tract; colon	A01.456.505.631; A03.556.500; A14.549; A11.329.228; A03.556.875; A04; A03.556.124.526.356; A03.556.249.249.356	periodontitis	C07.465.714.533	extracellular; encapsulated; unencapsulated; non-motile; direct physical contact; oral flora
Pseudomonas aeruginosa	skin; urinary tract; gastrointestinal tract; kidney; lung; blood	A17.815; A05.810; A03.556; A05.810.453; A04.411; A12.207.152; A15.145	inflammation; septicemia; skin diseases, infectious; soft tissue infections; gastroenteritis; urinary tract infection; pneumonia	C23.550.470; C01.539.757; C23.550.470.790.500; C01.539.800; C17.800.838; C01.539.820; C06.405.205; C01.539.895; C12.777.892; C13.351.968.892; C08.381.677; C08.730.610	wounds infection; extracellular; opportunity infection immunocompromised; encapsulated; motile; droplet contact; direct physical contact; airborne transmission; skin flora; respiratory flora
Respiratory Syncytial Virus	respiratory tract	A04	bronchiolitis; pneumonia	C08.127.446.135; C08.381.495.146.135;	acute infection; enveloped; cytoplasm; droplet contact; direct

				C08.730.099.135; C08.381.677; C08.730.610	physical contact; indirect physical contact
Severe Acute Respiratory Syndrome-Associated Coronavirus	respiratory tract	A04	severe acute respiratory syndrome	C02.782.600.550.200.750; C08.730.730	acute infection; enveloped; cytoplasm; viral droplet nuclei transmission; droplet contact; direct physical contact; blood
Shigella flexneri	gastrointestinal tract	A03.556	diarrhea; bacillary dysentery; gastroenteritis	C23.888.821.214; C01.252.400.310.229; C06.405.205.331.479; C06.405.469.300.479; C06.405.205	commensalism; extracellular; unencapsulated; non-motile; fecal-oral
Staphylococcus aureus	respiratory tract; skin; nose; hair follicles; perineum	A04; A17.815; A01.456.505.733; A04.531; A09.531; A10.272.497.500; A17.360.710; A17.815.250.500; A01.719	acne; impetigo; furuncles; furuncles; cellulitis; folliculitis; carbuncles; staphylococcal scalded skin syndrome; abscesses; sinusitis; pneumonia; meningitis; osteomyelitis; endocarditis; toxic shock syndrome; bacteremia; septicemia; skin diseases, infectious	C17.800.030.150; C17.800.271.125.200; C17.800.794.111; C01.252.410.868.820.504; C01.252.410.890.485; C01.252.825.770.360; C01.539.800.720.770.360; C17.800.838.765.770.360; C01.252.410.868.820.270; C01.252.825.770.270; C01.539.800.720.770.270; C17.800.838.765.770.270; C22.362.224; C01.252.410.868.820.270; C01.252.825.770.270; C01.539.800.720.770.270; C17.800.838.765.770.270; C22.362.224; C01.539.800.130; C01.539.830.200; C17.300.185; C23.550.470.756.200; C17.800.329.500; C01.252.410.868.820.270.200; C01.252.825.770.270.200;	wounds infection; extracellular; facultative anaerobic; beta-hemplytic; encapsulated; unencapsulated; non-motile; direct physical contact; vertical transmission; fecal-oral; mucosae flora; vaginal flora; conjunctival flora; hair follicles; external ear; gut flora; intestinal flora; oral flora; urogenital flora

				C01.539.800.720.770.270.200; C17.800.838.765.770.270.200; C01.252.410.868.820.770; C01.252.825.770.770; C01.539.800.720.770.770; C17.800.838.765.770.770; C01.539.830.025; C23.550.470.756.100; C08.460.692.752; C08.730.749; C09.603.692.752; C08.381.677; C08.730.610; C10.228.228.507; C10.228.566; C01.539.160.495; C05.116.165.495; C14.280.282; C01.539.757.800; C23.550.470.790.500.800; C23.550.835.900.712; C01.252.100; C01.539.757.100; C23.550.470.790.500.100; C01.539.757; C23.550.470.790.500; C01.539.800; C17.800.838	
Streptococcus agalactiae	gastrointestinal tract; mammary glands	A03.556; A01.236.249; A10.336.532	septicemia; bacterial meningitis; mastitis	C01.539.757; C23.550.470.790.500; C01.252.200.500; C10.228.228.180.500; C10.228.228.507.280; C13.703.844.603; C17.800.090.968	extracellular; facultative anaerobic; beta-hemplytic; encapsulated; unencapsulated; non-motile; vertical transmission; sexual contact; mucosae flora
Streptococcus gordonii	oral cavity; salivary pellicle	A01.456.505.631; A03.556.500; A14.549; A14.549.167.900.255.500	periodontitis; endocarditis, bacterial	C07.465.714.533; C01.252.300; C01.539.190.249; C14.260.249; C14.280.282.407	commensalism; extracellular; alpha-hemplytic; encapsulated; unencapsulated; non-motile; direct physical contact
Streptococcus pneumoniae	nasopharynx; lung; brain	A04.623.557; A14.724.557; A04.411; A08.186.211	pneumonia; pneumococcal infections;	C08.381.677; C08.730.610; C01.252.410.890.670;	extracellular; opportunity infection immunocompromised;

			sinusitis; otitis media; conjunctivitis; meningitis; bacteremia; septicemia; osteomyelitis; septic arthritis; pneumonia, bacterial; endocarditis, bacterial; peritonitis; pericarditis; cellulitis; brain abscess	C08.460.692.752; C08.730.749; C09.603.692.752; C09.218.705.663; C11.187.183; C10.228.228.507; C10.228.566; C01.252.100; C01.539.757.100; C23.550.470.790.500.100; C01.539.757; C23.550.470.790.500; C01.539.160.495; C05.116.165.495; C01.539.100; C05.550.114.099; C01.252.620; C08.381.677.540; C08.730.610.540; C01.252.300; C01.539.190.249; C14.260.249; C14.280.282.407; C01.539.463.600; C06.844.640; C14.280.720; C01.539.800.130; C01.539.830.200; C17.300.185; C23.550.470.756.200; C01.252.200.100; C01.323; C01.539.830.025.160; C10.228.140.116; C10.228.228.090; C10.228.228.180.100	alpha-hemplytic; encapsulated; unencapsulated; non-motile; respiratory droplets; droplet contact; respiratory flora
Streptococcus suis	lung; skin; joint; brain; nervous system	A04.411; A17.815; A02.835.583; A08.186.211; A08	pneumonia; meningitis; sepsis; endocarditis; deafness	C08.381.677; C08.730.610; C10.228.228.507; C10.228.566; C01.539.757; C23.550.470.790.500; C14.280.282; C09.218.458.341.186; C10.597.751.418.341.186; C23.888.592.763.393.341.186	facultative anaerobic; alpha-hemplytic; encapsulated; unencapsulated; non-motile; vector-borne transmission
Trypanosoma cruzi	blood; heart; esophagus; colon; peripheral	A12.207.152; A15.145; A07.541; A03.556.875.500;	chagas disease; chagas disease	C03.752.300.900.200; C03.752.300.900.200	obligate intracellular; intracellular infection; vector-borne

	nervous system	A03.556.124.526.356; A03.556.249.249.356; A08.800			transmission; indirect physical contact
Vaccinia Virus	cytoplasm	A11.284.430.214	smallpox	C02.256.743.826	acute infection; enveloped; cytoplasm; direct physical contact
Vesicular Stomatitis Virus	oropharynx	A04.623.603; A14.724.603			acute infection; enveloped; cytoplasm; saliva; droplet contact
Western Equine Encephalitis Virus	brain	A08.186.211	western equine encephalomyelitis	C02.081.355.677; C02.182.500.300.450.300; C02.290.450.250; C02.782.930.100.370.662; C10.228.228.210.150.300.450.800; C10.228.228.245.340.450.250; C10.228.228.291.323.662; C10.228.440.406.250	chronic infection; persistent infection; enveloped; cytoplasm; vector-borne transmission

Supplementary Table S3. The 36 enriched host transcriptional response-related infection attributes (HTR-IAs).

Rank Number	Enriched HTR-IA term/code	Term description	Number in total edges	Score	P value	FDR
1	facultative intracellular	Facultative Intracellular	15	0.739	$<10^{-5}$	-
2	intracellular infection	Intracellular Infection	55	0.534	$<10^{-5}$	-
3	B03.660	Proteobacteria	45	0.501	$<10^{-5}$	-
4	C01.252.400	Gram-negative Bacterial Infections	36	0.411	$<10^{-5}$	-
5	C08.381.677	Pneumonia	66	0.382	$<10^{-5}$	-
5	C08.730.610	Pneumonia	66	0.382	$<10^{-5}$	-
6	B03.440	Gram-negative Bacteria	91	0.346	$<10^{-5}$	-
7	droplet contact	Droplet Contact	55	0.342	$<10^{-5}$	-
8	C01.252	Bacterial Infections	91	0.323	$<10^{-5}$	-
9	unencapsulated	Unencapsulated	78	0.301	$<10^{-5}$	-
10	B03	Bacteria	210	0.297	$<10^{-5}$	-
11	encapsulated	Encapsulated	91	0.294	$<10^{-5}$	-
12	C08.381	Lung Diseases	78	0.274	$<10^{-5}$	-
13	C01	Bacterial Infections And Mycoses	153	0.249	$<10^{-5}$	-
14	C08	Respiratory Tract Diseases	105	0.247	$<10^{-5}$	-
15	C08.730	Respiratory Tract Infections	105	0.247	$<10^{-5}$	-
16	A04	Respiratory System	231	0.165	$<10^{-5}$	-
17	non-motile	Non-motile	78	0.259	$1.00*10^{-5}$	$6.74*10^{-5}$
18	C23	Pathological Conditions, Signs And Symptoms	91	0.240	$1.00*10^{-5}$	$6.74*10^{-5}$
19	C01.539.757	Bacteremia	36	0.356	0.0001	0.0003
20	A15	Hemic And Immune Systems	91	0.203	0.0002	0.0013
21	commensalism	Commensalism	10	0.594	0.0002	0.0013
22	A12	Fluids And Secretions	21	0.395	0.0007	0.0038
23	A03.556	Gastrointestinal Tract	171	0.126	0.0015	0.0079

Supplementary Table S4. Enriched infection attributes (IAs) identified for eight host transcriptional response (HTR) communities.

IA Type	Community	Enriched IAs	Term description	*P*-value
Infection tropism (implicated cell/tissue/organ)	3	A12	Fluids and Secretions	0.014
		A12.207	Body Fluids	0.044
	4	A08.663	Neurons	0.002
		A11	Cells	0.042
		A11.671	Neurons	0.002
	5	A02.835	Skeleton	0.023
		A02.835.583	Joints	0.023
		A17	Integumentary System	0.048
		A17.815	Skin	0.048
	7	A09	Sense Organs	0.045
	8	A01.456.505.631	Mouth	0.009
		A03.556.500	Mouth	0.009
		A14	Stomatognathic System	0.003
		A14.549	Mouth	0.018
Organisms	1	B01	Eukaryota	0.035
		B01.043	Alveolata	0.007
		B01.043.075	Apicomplexa	0.007
		B01.043.075.189	Coccidia	0.002
		B01.043.075.189.250	Eimeriida	0.002
		B01.043.075.189.250.150	Cryptosporidiidae	0.002
		B01.043.075.189.250.150.160	Cryptosporidium	0.002
	2	B04.820	RNA Viruses	0.020
		B04.820.455	Mononegavirales	0.048
		B04.909.777	RNA Viruses	0.020
		B04.909.777.455	Mononegavirales	0.048
	4	B04.280	DNA Viruses	0.003
		B04.280.382	Herpesviridae	0.023
		B04.280.382.100	Alphaherpesvirinae	0.002
		B04.280.382.100.750	Simplexvirus	0.002
		B04.909.204	DNA Viruses	0.003
		B04.909.204.382	Herpesviridae	0.023
		B04.909.204.382.100	Alphaherpesvirinae	0.002
		B04.909.204.382.100.750	Simplexvirus	0.002
	5	B04.280	DNA Viruses	0.024
		B04.909.204	DNA Viruses	0.024

		B04.909.204.210	DNA Tumor Viruses	0.023
		B04.909.574	Oncogenic Viruses	0.023
		B04.909.574.204	DNA Tumor Viruses	0.023
	6	B03	Bacteria	0.017
		B03.440	Gram-Negative Bacteria	0.014
		B03.440.400	Gram-Negative Aerobic Bacteria	0.007
		B03.440.400.425	Gram-Negative Aerobic Rods and Cocci	0.007
		B03.660	Proteobacteria	0.023
		B03.660.075	Betaproteobacteria	0.017
	7	B03.660	Proteobacteria	0.048
	8	B04.820.565	Picornaviridae	0.035
		B04.909.777.618	Picornaviridae	0.035
Manifestations of infectious diseases	1	C03	Parasitic Diseases	0.023
		C03.432	Intestinal Diseases, Parasitic	0.002
		C03.432.269	Cryptosporidiosis	0.002
		C03.701	Parasitic Diseases, Animal	0.002
		C03.701.688	Protozoan Infections, Animal	0.002
		C03.701.688.235	Cryptosporidiosis	0.002
		C03.752	Protozoan Infections	0.023
		C03.752.250	Coccidiosis	0.002
		C03.752.250.269	Cryptosporidiosis	0.002
		C03.752.625	Protozoan Infections, Animal	0.002
		C03.752.625.235	Cryptosporidiosis	0.002
		C06.405.469	Intestinal Diseases	0.035
		C06.405.469.452	Intestinal Diseases, Parasitic	0.002
		C06.405.469.452.269	Cryptosporidiosis	0.002
		C22	Animal Diseases	0.007
		C22.674	Parasitic Diseases, Animal	0.002
		C22.674.710	Protozoan Infections, Animal	0.002

		C22.674.710.235	Cryptosporidiosis	0.002
	3	C04	Neoplasms	0.044
	4	C02.256	DNA Virus Infections	0.048
	5	C02.256	DNA Virus Infections	0.016
		C02.256.466	Herpesviridae Infections	0.045
		C02.256.466.313	Epstein-Barr Virus Infections	0.008
		C02.256.466.313.400	Infectious Mononucleosis	0.008
		C10.597	Neurologic Manifestations	0.045
		C14	Cardiovascular Diseases	0.016
		C15.378	Hematologic Diseases	0.045
		C15.378.553	Leukocyte Disorders	0.008
		C15.378.553.381	Infectious Mononucleosis	0.008
		C15.604	Lymphatic Diseases	0.023
		C15.604.515	Lymphoproliferative Disorders	0.023
		C15.604.515.516	Infectious Mononucleosis	0.008
		C20.683	Immunoproliferative Disorders	0.045
		C20.683.515	Bacterial Infections and Mycoses	0.023
		C20.683.515.515	Sepsis	0.008
		C23.888.592	Pathological Conditions, Signs and Symptoms	0.023
	6	C01	Pathologic Processes	0.049
		C01.539.757	Bacteremia	0.015
		C23	Systemic Inflammatory Response Syndrome	0.014
		C23.550	Sepsis	0.034
		C23.550.470	Bacterial Infections	0.023
		C23.550.470.790	Gram-Negative Bacterial Infections	0.023

		C23.550.470.790.500	Tick-Borne Diseases	0.023
	7	C01.252	Suppuration	0.018
		C01.252.400	Suppuration	0.035
		C01.252.400.825	Endocarditis, Bacterial	0.023
		C01.539.830	Cardiovascular Infections	0.045
		C23.550.470.756	Endocarditis, Bacterial	0.045
	8	C01.252.300	Picornaviridae Infections	0.012
		C01.539.190	Enterovirus Infections	0.012
		C01.539.190.249	Mouth Diseases	0.012
		C02.782.687	Periodontal Diseases	0.035
		C02.782.687.359	Periodontitis	0.012
		C07.465	Conjunctival Diseases	0.018
		C07.465.714	Conjunctivitis	0.004
		C07.465.714.533	Cardiovascular Infections	0.004
		C11.187	Endocarditis, Bacterial	0.035
		C11.187.183	Endocarditis, Bacterial	0.035
		C14.260	Bacterial Infections and Mycoses	0.012
		C14.260.249	Sepsis	0.012
		C14.280.282.407	Pathological Conditions, Signs and Symptoms	0.012
Important laboratory and clinical infection characteristics	1	obligate intracellular	obligate intracellular	0.023
		opportunity infection immunocompromised	opportunity infection immunocompromised	0.048
	3	cancer bacteria	cancer bacteria	0.019
		chronic infection	chronic infection	0.038
		facultative intracellular	facultative intracellular	0.044
		intracellular infection	intracellular infection	0.036
		oncogenic infection	oncogenic infection	0.004
	4	enveloped	enveloped	0.042
		latent infection	latent infection	0.023

	5	nucleus	nucleus	0.048
	6	encapsulated	encapsulated	0.014
		extracellular	extracellular	0.048
		respiratory flora	respiratory flora	0.048
		skin flora	skin flora	0.048
	7	encapsulated	encapsulated	0.018
		gut flora	gut flora	0.008
		intestinal flora	intestinal flora	0.008
	8	alpha-hemplytic	alpha-hemplytic	0.035
		commensalism	commensalism	0.009
		non-motile	non-motile	0.033

Supplementary Table S5. Enriched bioprocesses identified for eight host transcriptional response (HTR) communities.

Regulation direction	Community	Enriched Bioprocess	*P*-value
up	2	CATABOLIC_PROCESS	0.0018
		CELLULAR_CATABOLIC_PROCESS	0.0007
		CELLULAR_MACROMOLECULE_CATABOLIC_PROCESS	0.0067
		ESTABLISHMENT_OF_CELLULAR_LOCALIZATION	0.0085
		ESTABLISHMENT_OF_PROTEIN_LOCALIZATION	0.0067
		INTRACELLULAR_PROTEIN_TRANSPORT	0.0045
		PROTEIN_LOCALIZATION	0.0067
		PROTEIN_TARGETING	0.0067
		PROTEIN_TRANSPORT	0.0020
	3	CELLULAR_DEFENSE_RESPONSE	0.0047
		DEFENSE_RESPONSE	0.0038
		IMMUNE_RESPONSE	0.0009
		IMMUNE_SYSTEM_PROCESS	0.0019
		INFLAMMATORY_RESPONSE	0.0038
		I_KAPPAB_KINASE_NF_KAPPAB_CASCADE	0.0071
		NEGATIVE_REGULATION_OF_APOPTOSIS	0.0009
		NEGATIVE_REGULATION_OF_DEVELOPMENTAL_PROCESS	0.0009
		NEGATIVE_REGULATION_OF_PROGRAMMED_CELL_DEATH	0.0026
		POSITIVE_REGULATION_OF_I_KAPPAB_KINASE_NF_KAPPAB_CASCADE	0.0019
		POSITIVE_REGULATION_OF_MULTICELLULAR_ORGANISMAL_PROCESS	0.0043
		POSITIVE_REGULATION_OF_SIGNAL_TRANSDUCTION	0.0001
		PROTEIN_KINASE_CASCADE	0.0071
		REGULATION_OF_DEVELOPMENTAL_PROCESS	0.0019
		REGULATION_OF_I_KAPPAB_KINASE_NF_KAPPAB_CASCADE	0.0009
	6	CAMP_MEDIATED_SIGNALING	0.0020
		CELL_CELL_SIGNALING	0.0017

		CYCLIC_NUCLEOTIDE_MEDIATED_SIGNALING	0.0001
		G_PROTEIN_COUPLED_RECEPTOR_PROTEIN_SIGNALING_PATHWAY	0.0043
		G_PROTEIN_SIGNALING_COUPLED_TO_CAMP_NUCLEOTIDE_SECOND_MESSENGER	0.0007
		G_PROTEIN_SIGNALING_COUPLED_TO_CYCLIC_NUCLEOTIDE_SECOND_MESSENGER	0.0001
		NEUROLOGICAL_SYSTEM_PROCESS	0.0059
		REGULATION_OF_BIOLOGICAL_QUALITY	0.0045
		SECOND_MESSENGER_MEDIATED_SIGNALING	0.0004
	7	G_PROTEIN_COUPLED_RECEPTOR_PROTEIN_SIGNALING_PATHWAY	0.0029
down	5	G_PROTEIN_COUPLED_RECEPTOR_PROTEIN_SIGNALING_PATHWAY	0.0025
		SENSORY_PERCEPTION	0.0082
	6	CELL_CYCLE_GO_0007049	0.0036
		CELL_CYCLE_PROCESS	0.0011
		CHROMOSOME_ORGANIZATION_AND_BIOGENESIS	0.0085
		DNA_DEPENDENT_DNA_REPLICATION	0.0005
		DNA_METABOLIC_PROCESS	0.0059
		DNA_REPAIR	0.0001
		MITOSIS	0.0021
		M_PHASE	0.0036
		M_PHASE_OF_MITOTIC_CELL_CYCLE	0.0004
		ORGANELLE_ORGANIZATION_AND_BIOGENESIS	0.0092
		RESPONSE_TO_DNA_DAMAGE_STIMULUS	7.93E-06
		RESPONSE_TO_ENDOGENOUS_STIMULUS	0.0021
	8	INTRACELLULAR_TRANSPORT	0.0020
		MRNA_METABOLIC_PROCESS	0.0047
		RIBONUCLEOPROTEIN_COMPLEX_BIOGENESIS_AND_ASSEMBLY	0.0069
		RNA_PROCESSING	0.0069

Supplementary Table S6. Gene expression profiles datasets used for generating external pathogen query signatures.

Pathogen type	Organisms	Strain[a]	Infection model			GSE NO.	Platform	Time course	Reference
			in vitro/ in vivo	cell or tissue resource	cell or tissue types				
bacteria	*Salmonella enterica* Subspecies *typhimurium*	SL1344	in vitro	Homo sapiens	human monocytic tissue culture cells	GSE503	GPL178	4h	Detweiler CS, et al. PNAS 2001.
							GPL181	4h	
							GPL182	4h	
							GPL183	3h	
	Burkholderia cepacia	Two clinical strains isolated from the sputum of an individual with CF, AD2A and AD15B	in vivo	Homo sapiens	human alveolar macrophages obtained from bronchoalveolar lavage	GSE12245	GPL80	—	Worgall S, et al. Infect Immun, 2005.
	Enterococcus faecalis	—	in vitro	Homo sapiens	human urothelial cells (HUC from ATCC)	GSE5988	GPL3463	0-10 h	Dozmorov MG, et al. BMC Bioinformatics. 2007
	Streptococcus pyogenes	wild-type strain MGAS5005	in vivo	Cynomolgus macaques	tonsil swabs	GSE20262	GPL96	0-32 days	Shea PR, et al. PNAS, 2010.
	Treponema denticola	ATCC 35404	in vivo	mice	calvarial bones and overlying soft tissues	GSE19855	GPL339	3 days	Bakthavatchalu V, et al. Mol Oral Microbiol 2010.
virus	Influenza A Virus	A/Netherland/219/2003 (H7N7), A/Vietnam/1203/2004 (H5N1), A/Panama/2007/1999 (H3N2) A/Anhui/01/13 (H7N9)	in vitro	Homo sapiens	human lung epithelial cell (Calu-3 cells)	GSE49840	GPL17077	3, 7, 12 and 24 hours	Josset L,et al. MBio, 2014
	human parainfluenza virus (hPIV)	hPIV1 wild type	in vitro	Homo sapiens	A549 human respiratory epithelial cells	GSE12644	GPL6480	6, 12, 24, and 48 hours	Boonyaratanakornkit JB, et al. J Virol, 2009
	Human metapneumovirus (hMPV)	—	in vitro	Homo sapiens	A549 human respiratory epithelial cells	GSE8961	GPL	6, 12, 24, 48, or 72 hours	Bao X, et al. Virology, 2008.

"—", no specific declaration on the designated information in the dataset descriptions.

Supplementary Data (provided as independent files)

Supplementary Data S1. Spearman correlation coefficients between individual PRLs.

Supplementary Data S2. mPRLs of 50 pathogens.

Supplementary Data S3. Spearman correlation coefficients between individual PRLs and mPRLs.

Supplementary Data S4. Association Scores between 50 mPRLs generated with different signature sizes.

Supplementary Data S5. Association Scores between 50 mPRLs and corresponding P values and FDR values.

Supplementary Data S6. Enrichment Scores of GO BP signatures in 50 mPRLs and corresponding P values and FDR values.

Supplementary Data S7. Association Scores between Protebacteria.

Supplementary Data S8. Association Scores between external query signatures and 50 mPRLs.

Supplementary Signatures (provided as independent files)

Supplementary Signature S1. Signature of *Salmonella typhimurium* from GSE503 (GPL178).

Supplementary Signature S2. Signature of *Salmonella typhimurium* from GSE503 (GPL181).

Supplementary Signature S3. Signature of *Salmonella typhimurium* from GSE503 (GPL182).

Supplementary Signature S4. Signature of *Salmonella typhimurium* from GSE503 (GPL183).

Supplementary Signature S5. Signature of *Salmonella typhimurium* (PHOP mutant) from GSE503 (GPL183).

Supplementary Signature S6. Signature of *Burkholderia cepacia* from GSE12245.

Supplementary Signature S7. Signature of *Enterococcus faecalis* from GSE5988.

Supplementary Signature S8. Signature of *Streptococcus pyogenes* from GSE20262.

Supplementary Signature S9. Signature of *Treponema denticola* from GSE19855 (tissue).

Supplementary Signature S10. Signature of *Treponema denticola* from GSE19855 (bone).

Supplementary Signature S11. Signature of Influenza A virus from GSE49840 (H7N9).

Supplementary Signature S12. Signature of Influenza A virus from GSE49840 (H5N1).

Supplementary Signature S13. Signature of Influenza A virus from GSE49840 (H7N7).

Supplementary Signature S14. Signature of Influenza A virus from GSE49840 (H3N2).

Supplementary Signature S15. Signature of Human Metapneumovirus from GSE8961.

Supplementary Signature S16. Signature of Human Parainfluenza virus from GSE12664 (WT).
